# The effect of prior healthcare employment on the wages of registered nurses

**DOI:** 10.1186/s12913-016-1667-0

**Published:** 2016-08-19

**Authors:** Byung-Kwang Yoo, Minchul Kim, Tzu-Chun Lin, Tomoko Sasaki, Debbie Ward, Joanne Spetz

**Affiliations:** 1 Department of Public Health Sciences, University of California, Davis, School of Medicine, One Shields Ave., Medical Sciences 1C, Davis, CA 95616 USA; 2 Center for Outcomes Research, Department of Internal Medicine, University of Illinois College of Medicine at Peoria, One Illini Drive, Peoria, IL 61656 USA; 3 Department of Statistics, Graduate Group in Biostatistics, University of California, Davis, Mathematical Sciences Building 4118, One Shields Avenue, Davis, CA 95616 USA; 4 Independent Consultant, 1766 Crittenden Rd, Rochester, NY 14623 USA; 5 Betty Irene Moore School of Nursing, University of California, Davis, 4610 X Street, #4202, Sacramento, CA, 95817 USA; 6 Philip R. Lee Institute for Health Policy Studies & Healthforce Center, University of California, San Francisco, 3333 California Street, Suite 265, San Francisco, CA 94118 USA

**Keywords:** Registered Nurse, Wage, Salary, Prior Employment, Education of Registered Nurse

## Abstract

**Background:**

The proportion of registered nurses (RNs) with employment in health-related positions before their initial RN education has increased in the past two decades. Previous research found that prior health-related employment is positively associated with RN workforce supply, potentially due to the wage differences based on different career paths. This study’s objective is to test the hypotheses that prior health-related employment is associated with differences in starting wages and with different rates of wage growth for experience as an RN.

**Methods:**

We conducted a cross-sectional analysis using the 2008 National Sample Survey of Registered Nurses (NSSRN) linked with county-level variables from the Area Health Resource File. We estimated a Heckman model where the second-stage equation’s outcome variable was the logarithm of the RN hourly wage, accounting for the self-selection of working or not working as an RN (i.e., the first-stage equation’s outcome variable). Key covariates included interaction terms between years of experience, experience squared, and six categories of prior health-related employment (manager, LPN/LVN, allied health, nursing aide, clerk, and all other healthcare positions). Additional covariates included demographics, weekly working hours, marital status, highest nursing degree, and county-level variables (e.g., unemployment rate). We estimated the marginal effect of experience on wage for each type of prior health-related employment, conducting separate analyses for RNs whose initial education was a Bachelor of Science in Nursing (BSN) (unweighted *N* = 10,345/weighted *N* = 945,429), RNs whose initial education was an Associate degree (unweighted *N* = 13,791/weighted *N* = 1,296,809), and total population combining the former groups (unweighted *N* = 24,136/weighted *N* = 2,242,238).

**Results:**

Prior health-related employment was associated with higher wages, with the strongest wage differences among BSN-educated RNs. Among BSN-educated RNs, previous employment as a health care manager, LPN/LVN, or nursing aide produced statistically higher starting wages ($1.72-$3.86 per hour; $3400–$7700 per year; *p* = 0.006–0.08). However, experience-based wage growth was lower for BSN-educated RNs previously employed as allied health workers, LPN/LVNs, or nursing aides. Among Associate degree-educated RNs, wage difference was not observed except for higher initial wage for RNs with previous employment as LPN/LVNs.

**Conclusions:**

Prior employment in health-related positions was associated with both starting salary and experience-based wage growth among BSN-educated RNs. The higher wage return for those with a BSN may motivate non-RN healthcare workers to seek a BSN in their transition to RN jobs, which could help advancement toward the 80 % BSN workforce recommended by the U.S. Institute of Medicine.

**Electronic supplementary material:**

The online version of this article (doi:10.1186/s12913-016-1667-0) contains supplementary material, which is available to authorized users.

## Background

The backgrounds of registered nurses (RNs) have become more diverse in the past two decades. For instance, the proportion of RNs with prior employment in health-related positions before completing their initial RN education was 67.2 % in 2008, having increased steadily from 28.8 % in 1992 [1]. Previous research found that prior health-related employment is positively associated with RN workforce supply [2]. Specifically, prior healthcare employment in a lower-wage occupation (e.g., allied health, nursing aide, and clerk) was positively associated with RN workforce participation. Additionally, prior health-related employment in higher wage occupations (e.g., health care manager and licensed practical nurses or licensed vocational nurses (LPN/LVN)) was positively associated with working longer hours. Such positive associations with RN workforce supply could be partly explained by the wage differences between RNs with and without prior health-related employment as well as by the global wage difference between RNs and non-RN health related occupations.

For RNs with prior health-related employment, a relevant research question is whether having prior experience increases human capital and therefore raises the marginal product of nurse labor, which, in competitive labor markets, raises the wage. This is because employers are expected to pay higher wages for attracting RNs with better clinical expertise, which contributes both to hospital competitiveness and ability to maximize returns in value-based payment systems such as recent performance-based reimbursement reforms implemented in the United States (U.S.) [3].

To our knowledge, no study has explored the effect of prior non-RN healthcare employment on RN wages. The empirical findings on this potential effect would be appreciated by three categories of readers. First, for those considering an RN career after their current healthcare occupation, this study’s findings would help determine their career plan by providing more detailed wage information other than the national-average of an RN wage. Second, for RN employers such as hospitals and long-term care facilities, this study’s results would provide data about how the marginal product of RN labor is valued in a nationally representative RN sample. Finally, for national leaders in nursing and healthcare providers, this study’s results clarify whether the current wage level and the expected wage growth could motivate or discourage (a) a certain type of initial RN education pursued and (b) transition from a non-RN healthcare occupation to an RN licensure (that may affect the long-term RN labor supply).

Prospective nurses can enter the profession in the U.S. after completing an RN education program and passing the NCLEX-RN, which is a national licensure examination. More than half of new RN graduates (53 %) complete an Associate degree program at a community or private college [4]. About 39% graduate with a Bachelor of science in nursing (BSN) and less than 4% complete a graduate degree program designed for initial RN education [4–6]. Less than 2% of new RNs complete a diploma program based at a hospital [4]. The U.S. Institute of Medicine Committee on the Future of Nursing recommended that, by 2020, 80% of RNs hold a BSN or higher degree; this goal can be attained by increasing the share of new graduates with a BSN and by supporting educational advancement (i.e., completing a BSN after licensure) [7].

Thus, the present study aims to test three specific hypotheses: (1) RNs with prior healthcare employment will have different starting wages than RNs without prior healthcare employment (control group); (2) RNs with prior healthcare employment will have different experience-based wage growth than the control group; and (3) RNs with prior healthcare employment will have different starting wages and different experience-based wage growth, depending on their initial RN education, either BSN or Associate degree. Hypothesis 1 was generated by the assumption that employers will pay higher wages to attract RNs with better clinical expertise in a competitive labor market. Additionally, evidence supporting Hypothesis 1 would be consistent with prior research that finds greater employment activity among those with prior health care experience, since the higher starting wage associated with prior employment may be motivation to keep working as an RN [2]. Hypothesis 2 was derived from the assumption that having prior experience in health care enables one to develop RN skills on the job more efficiently, i.e., more efficient human capital accumulation. For instance, if you already know the basics of how an electronic health record (EHR) functions, you can focus on developing clinical RN skills rather than trying to develop EHR and clinical skills simultaneously. Hypothesis 3 was derived from prior research that found different starting wages and different experience-based wage growth for nurses with initial BSN education versus Associate degree education [3, 8]. There may be combined differences in wage trajectories based on both initial RN education and prior health care employment, and these differences may interact.

## Methods

### Datasets

We analyzed the National Sample Survey of Registered Nurses (NSSRN) from 2008, which is a nationally-representative survey of the RN population [1]. It targeted all RNs holding active nursing licenses as of March 2008 regardless of their employment status. We linked these data with county-level variables in the Area Health Resource File (AHRF) [9]. As a cross-sectional dataset, NSSRN data was collected every 4 years, from 1980 to 2008, by the Health Resources and Services Administration (HRSA) of the U.S. Department of Health and Human Services [3, 10]. This survey contains detailed questions about nursing and non-nursing education, employment setting and job title, hours worked per week and per year, own and household earnings, and demographic characteristics [3, 11]. All NSSRN data such as marital status and work status indicate the status in the collection year of 2008 [12]. Following the recommendation of the 2008 NSSRN data codebook [12], we weighted samples to reduce sampling error, which causes sample estimates to differ from the true total RN population parameter values [1]. In other words, unweighted samples, who responded to the NSSRN survey, were converted to weighted samples that represent the true total RN population at the U.S. national level.

### Sample

Out of 33,352 actively licensed RNs in the NSSRN 2008 public use data file, we excluded 1966 RNs who responded to the survey and resided outside the U.S. (the remaining unweighted *N* = 31,386/weighted *N* = 2,903,220). We additionally excluded RNs whose initial nursing education was at the diploma or graduate (Master’s or Doctorate) level, because both are now relatively uncommon. For instance, the former group proportion is only 3.7 % and the latter group proportion is 0.4 % among the newly graduated RNs in the 2008 NSSRN data. Distinguishing two types of entry-level RN education, we made separate analyses for each of the two study populations: RNs whose initial education was a BSN (unweighted *N* = 10,345/weighted *N* = 945,429) and RNs whose initial education was an Associate degree (unweighted *N* = 13,791/weighted *N* = 1,296,809). Combining these two types of entry-level RN education to create the “total population” (unweighted *N* = 24,136/weighted *N* = 2,242,238), we also analyzed this total population. All dollar values in this study are 2008 U.S. dollar values. All analyses used STATA version 12 [13].

### Statistical analyses

Our primary analysis examined the effect of prior healthcare employment on wage over years of working experience as an RN. Wages are observed only for employed RNs (i.e., the effective sample size of unweighted *N* = 21,225/weighted *N* =1,949,625), who are a self-selected group, and thus a simple analysis of wages will not reflect the effects of human capital variables on a randomly selected nurse. To account for this self-selection, we estimated a Heckman model in which the first-stage equation’s dependent variable was a dichotomous variable indicating whether a subject was employed or self-employed in nursing, i.e., working or not as a RN. The covariates uniquely included in the first-stage equation (i.e., excluded from the second-stage equation) were other household income, age (five categories), student status (full-time, part-time, or no student), children at home (four categories), and county-level characteristics (uninsurance rate and unemployment rate). These county-level variables were included as a proxy for macro economy in a subject’s county, which is assumed to affect the availability of alternative non-RN positions.

Another set of covariates included in both the first-stage equation and the second-stage equation were six categories of prior healthcare employment (manager, LPN/LVN, allied health, nursing aide, clerk, and all other healthcare positions), race, gender, marital status, highest nursing degree, county-level characteristics (primary care practitioners per 1000 population; and medical, surgical and other specialists per 1000 population as a proxy for healthcare providers’ demand for RNs), indicators for 50 states (and Washington DC), and urban area. These Heckman models were justified by the statistically significant rho, reported in the bottom row of Table 2.

In this Heckman model, the second-stage equation’s outcome variable was the logarithm of the RN hourly wage. Following previous research [3, 8, 14], total earnings from all nursing jobs were divided by the number of annual hours worked for all nursing jobs in order to calculate the RN hourly wage. The natural logarithm of the wage was used for the normal distribution approximation. The second-stage equation’s covariates are listed in Table 2, including the work status (part-time, full-time with overtime, or full time without overtime), work setting (hospital, nursing home or other), years of RN experience, experience squared, the six categories of prior healthcare employment and interaction terms between these six categories of prior healthcare employment and years of RN experience (or experience squared). The six categories of prior healthcare employment tested the first hypothesis that RNs with prior healthcare employment have a different starting wage than those without prior healthcare employment.

**Fig. 1:**
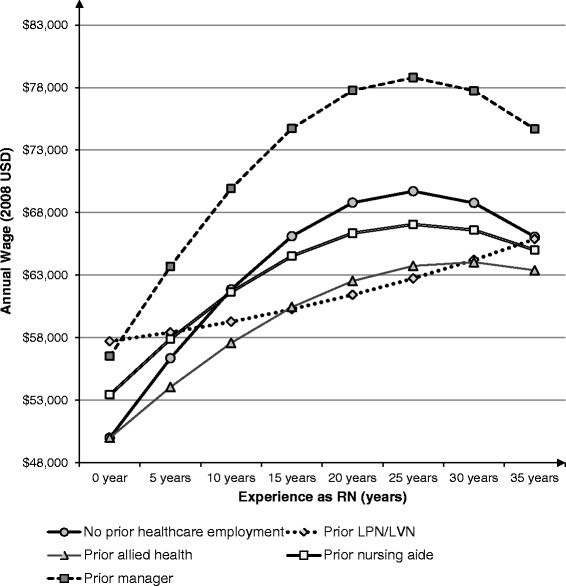
Expected annual wage^a^ over experience as registered nurses (RNs)^b^ [years] with various prior health-related employment^c^. **a** The expected annual wage [2008 US dollars (USD)] was calculated for every 5 years based on the mean values of covariates as follows: Expected RN hourly wage × 40 h × 50 weeks, derived from 2008 National Sample Survey of Registered Nurses (NSSRN) data. **b** Registered nurses (RN)s who started their initial nursing education with a Bachelor’s degree only (unweighted *N* = 10,345; weighted *N* = 945,429). **c** Prior employment includes manager, licensed practical nurses/licensed vocational nurses (LPN/LVN), allied health, and nursing aide

**Fig. 2:**
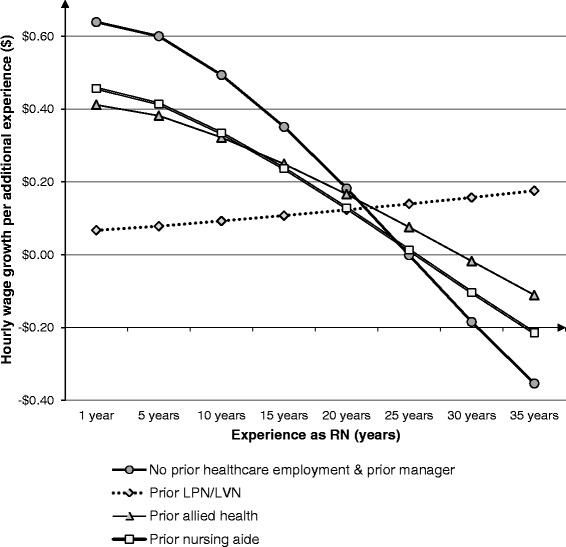
Hourly wage growth rate^a^ over years of experience as RNs^b^ with various prior health-related employment^c^. **a** Hourly wage growth-rate [2008 US dollars (USD)] was derived from 2008 National Sample Survey of Registered Nurses (NSSRN) data. **b** Registered nurses (RN)s who started their initial nursing education with a Bachelor’s degree only (unweighted *N* = 10,345; weighted *N* = 945,429). **c** Prior employment includes licensed practical nurses/licensed vocational nurses (LPN/LVN), allied health, and nursing aide

Experience was calculated by subtracting the year of first RN license from 2008 [3, 8, 14]. In order to reflect the effect of the non-working period on experience, one year was subtracted for RNs who left nursing for one or more years since becoming an RN (unweighted 11.7 % of sample), and 0.5 year was subtracted for RNs who are recent graduates and could not have left nursing for one or more years (unweighted 0.9 % of sample). The interaction terms between prior employment and experience (and experience squared) tested the second hypothesis that RNs with prior healthcare employment have different experience-based wage growth than RNs without prior healthcare employment. We estimated the marginal effect of experience for each type of prior healthcare employment at 5, 10, 15, 20, 25, 30 and 35 years of experience for creating Figs. 1 and 2.

We conducted sensitivity analyses to determine the model specifications of the experience variable, which were reported in Tables S1-S3 (available in Additional files 1, 2 and 3).

## Results

The summary statistics of the study subjects are presented in Table 1. The mean hourly wage was $31.14 and mean RN experience was 15.85 years among our total population, with some variation depending on the initial RN education. About one fourth (27.2 %; unweighted count = 5506 among the total population’s working sample shown in the bottom panel of Table 1) of RNs did not have any prior healthcare employment. This proportion was lower among Associate degree RNs (23.2 %). The six categories of prior healthcare employment are listed in descending order according to hourly wage as of May 2008 [15] : manager ($43; unweighted count = 395), LPN/LVN ($19; unweighted count = 3605), allied health ($14–16; unweighted count = 2548), clerk ($12.42; unweighted count = 661), nursing aide ($11.84; unweighted count = 7506), and all other healthcare positions (specific wage was not indicated due to combining multiple positions; unweighted count = 1004). The most common type of prior healthcare employment was nursing aide; 29 % to 43 % (depending on initial RN education type) of RNs worked as an aide before graduating from their initial RN education. Other common pre-nursing healthcare employment included LPN/LVN (5.0 %–23.8 %) and allied health (10.1 %–13.7 %).

**Table 1:** Demographic Characteristics of Working Registered Nurses (RNs) in 2008 NSSRN^a^ Data

Target population	Total Population^b^ (Initial Bachelor and Associate degree)		Initial Bachelor degree		Initial Associate degree	
Un-weighted sample size	Un-weighted *N* = 24,136	Un-weighted *N* = 21,225	Un-weighted *N* = 10,345	Un-weighted *N* = 8,992	Un-weighted *N* = 13,791	Un-weighted *N* = 12,223
Weighted sample size	Weighted *N* = 2,242,238	Weighted *N* = 1,949,625	Weighted *N* = 945,429	Weighted *N* = 812,458	Weighted *N* = 1,296,809	Weighted *N* = 1,137,166
Sample group	All sample (selection equation)	Working sample (wage equation)	All sample (selection equation)	Working sample (wage equation)	All sample (selection equation)	Working sample (wage equation)
I. Continuous variables	Weighted Mean (SD)					
RN hourly wage^c^ [$]	n/a (n/a)	31.14 (12.68)	n/a (n/a)	32.90 (13.65)	n/a (n/a)	29.88 (11.78)
Log of RN hourly wage (Outcome variable)	n/a (n/a)	3.37 (0.36)	n/a (n/a)	3.43 (0.36)	n/a (n/a)	3.33 (0.35)
RN Experience (in years)	n/a (n/a)	15.85 (10.68)	n/a (n/a)	16.44 (11.19)	n/a (n/a)	15.43 (10.29)
Medical, surgical and other specialists per 1,000 population	1.14 (0.87)	1.16 (0.88)	1.28 (0.91)	1.30 (0.92)	1.03 (0.83)	1.05 (0.84)
Primary care practitioners per 1,000 population	0.32 (0.15)	0.32 (0.15)	0.32 (0.15)	0.32 (0.15)	0.32 (0.15)	0.32 (0.15)
Log of other income	9.18 (3.56)	n/a (n/a)	9.34 (3.53)	n/a (n/a)	9.06 (3.57)	n/a (n/a)
Uninsured rate	14.45 (4.44)	n/a (n/a)	14.41 (4.57)	n/a (n/a)	14.49 (4.35)	n/a (n/a)
Unemployment rate	5.72 (1.53)	n/a (n/a)	5.58 (1.42)	n/a (n/a)	5.83 (1.59)	n/a (n/a)
II. Categorical variables	Weighted Percent (%)					
Prior healthcare position						
Manager	(1.8)	(1.7)	(1.3)	(1.1)	(2.2)	(2.2)
LPN/LVN^d^	(15.8)	(16.0)	(5.0)	(5.0)	(23.6)	(23.8)
Allied health	(11.9)	(12.2)	(10.0)	(10.1)	(13.4)	(13.7)
Nursing aide	(34.8)	(35.0)	(43.0)	(43.3)	(28.9)	(29.1)
Clerk	(3.2)	(3.3)	(2.7)	(2.8)	(3.6)	(3.6)
Other	(4.7)	(4.6)	(5.0)	(4.9)	(4.5)	(4.4)
No prior health care job	(27.8)	(27.2)	(33.2)	(32.9)	(23.8)	(23.2)
Gender						
Male	(7.3)	(7.6)	(6.9)	(7.1)	(7.6)	(8.0)
Female	(92.7)	(92.4)	(93.1)	(92.9)	(92.4)	(92.0)
Marital Status						
Married	(74.3)	(73.9)	(75.3)	(74.5)	(73.6)	(73.5)
Single	(25.7)	(26.1)	(24.7)	(25.5)	(26.4)	(26.5)
Race						
White	(82.3)	(81.5)	(78.5)	(77.4)	(85.0)	(84.3)
Other	(17.8)	(18.6)	(21.5)	(22.6)	(15.0)	(15.7)
Work Status						
Part-time	n/a	(23.4)	n/a	(25.7)	n/a	(21.8)
Full-time, no overtime	n/a	(41.2)	n/a	(40.3)	n/a	(41.7)
Full-time, with overtime	n/a	(35.4)	n/a	(34.0)	n/a	(36.4)
Work setting						
Hospital	n/a	(63.7)	n/a	(65.1)	n/a	(62.7)
Nursing home	n/a	(5.3)	n/a	(3.4)	n/a	(6.6)
Other setting	n/a	(31.1)	n/a	(31.6)	n/a	(30.7)
Highest nursing education						
Diploma	n/a	n/a	n/a	n/a	n/a	n/a
Associate degree	(45.6)	(46.1)	n/a	n/a	(78.9)	(79.1)
Bachelor	(41.7)	(41.4)	(79.3)	(79.7)	(14.3)	(14.0)
Master's	(12.7)	(12.5)	(20.7)	(20.3)	(6.9)	(6.9)
Working or not (Selection variable)						
Working	(87.0)	n/a	(85.9)	n/a	(87.7)	n/a
Not working	(13.1)	n/a	(14.1)	n/a	(12.3)	n/a
Grouped age						
<30	(11.0)	(12.0)	(15.1)	(16.6)	(7.9)	(8.7)
30–39	(23.3)	(24.4)	(25.9)	(26.6)	(21.4)	(22.7)
40–49	(28.5)	(29.4)	(27.1)	(27.7)	(29.4)	(30.5)
50–59	(27.9)	(27.6)	(24.3)	(23.7)	(30.5)	(30.5)
> = 60	(9.4)	(6.7)	(7.5)	(5.4)	(10.8)	(7.6)
Student enrollment						
Full-time student	(3.3)	(3.3)	(3.3)	(3.2)	(3.2)	(3.4)
Part-time student	(5.5)	(5.9)	(4.4)	(4.8)	(6.3)	(6.7)
Not a student	(91.3)	(90.8)	(92.3)	(92.0)	(90.5)	(90.0)
Children at home						
Children under 6 years	(10.7)	(11.0)	(13.5)	(13.8)	(8.6)	(9.0)
All children 6–18 years	(28.9)	(29.7)	(28.7)	(29.2)	(29.1)	(30.1)
Both under and over 6 years	(7.6)	(7.7)	(8.4)	(8.4)	(7.0)	(7.3)
No children at home	(52.8)	(51.6)	(49.3)	(48.7)	(55.3)	(53.7)
Urban/Rural						
Urban	(81.2)	(80.8)	(87.5)	(87.3)	(76.5)	(76.1)
Rural	(18.8)	(19.2)	(12.5)	(12.7)	(23.5)	(23.9)
III. Count of Prior Healthcare Position^d^		Weighted Count (Unweighted Count)				
Manager	40,583 (461)	33,694 (395)	12,056 (136)	8,809 (105)	28,527 (325)	24,885 (290)
LPN/LVN^e^	353,334 (4,033)	311,253 (3,605)	46,782 (564)	40,783 (498)	306,553 (3,469)	270,470 (3,107)
Allied health	267,431 (2,849)	237,609 (2,548)	94,316 (1,028)	81,849 (906)	173,114 (1,821)	155,760 (1,642)
Nursing aide	780,764 (8,470)	682,037 (7,506)	406,120 (4,563)	351,355 (4,010)	374,643 (3,907)	330,682 (3496)
Clerk	72,290 (753)	64,315 (661)	25,453 (277)	22,881 (247)	46,836 (476)	41,434 (414)
Other	105,368 (1,163)	90,257 (1,004)	46,869 (539)	39,890 (462)	58,499 (624)	50,367 (542)
No prior health care job	622,469 (6,407)	530,461 (5,506)	313,832 (3,238)	266,892 (2,764)	308,637 (3,169)	263,568 (2,742)

^a^NSSRN: nationally representative National Sample Survey of Registered Nurses

^b^All actively licensed RNs in the NSSRN 2008 public use data file, excluding RNs who resided outside the United States and RNs whose initial nursing education was at the diploma or graduate (Master’s or Doctorate) level

^c^RN hourly wage was calculated by adding the salaries from all nursing jobs, then dividing by the number of annual hours worked for all nursing jobs. The natural logarithm of the wage was used to make the distribution approximately normal

^d^Count data for other variables are summarized in Table S4 available in Additional file 4

^e^LPN/LVN: Licensed Practical Nurses/Licensed Vocational Nurses

Multivariate regression analysis supported the first hypothesis regarding the effect of prior healthcare employment on initial wage, as presented in Table 2. Initial wages were higher for RNs with prior employment as a manager, LPN/LVN, or nursing aide among our total population and subpopulation whose initial education was a BSN (BSN-educated RNs). Among RNs whose initial education was an Associate degree (Associate-educated RNs), higher initial wage was less prominent and found only with previous employment as a LPN/LVN. The coefficient for prior manager employment (0.095, *p* < 0.05) equates to a starting wage that was $2.48 higher among the total population. The initial hourly wages of BSN-educated nurses with prior LPN/LVN employment were higher by $3.86 (coefficient = 0.143, *p* < 0.05) and those with prior nursing aide employment were higher by $1.72 (coefficient = 0.066, *p* < 0.01). For Associate-educated RNs with prior LPN/LVN employment, the initial hourly wage was higher by $1.12 (coefficient = 0.042, *p* = 0.096).

**Table 2:** Determinants of RN hourly wage^‡^ based on Heckman model^§^ in 2008 NSSRN^†^ data

Dependent variable: Log of RN Hourly Wage	Total Population^a^ (Initial Bachelor and Associate degree)		Initial Bachelor degree		Initial Associate degree	
	Weighted *N* = 2,242,238		Weighted *N* = 945,429		Weighted *N* = 1,296,809	
	Un-weighted *N* = 24,136		Un-weighted *N* = 10,345		Un-weighted *N* = 13,791	
Explanatory variable (Reference category)	Coefficient	*P*-value	Coefficient	*P*-value	Coefficient	*P*-value
Experience^b^	0.023	0.001***	0.027	0.001***	0.018	0.001***
Experience Square	−4.52E-04	0.001***	−0.001	0.001***	−3.44E-04	0.001***
Prior healthcare job (no prior healthcare job)						
Manager	0.095	0.026**	0.123	0.083*	0.059	0.287
LPN/LVN	0.076	0.001***	0.143	0.034**	0.042	0.096*
Allied health	0.006	0.787	0.050	0.180	−0.034	0.205
Nursing aide	0.041	0.019**	0.066	0.006***	0.013	0.591
Clerk	0.007	0.842	0.022	0.670	−0.019	0.659
Other	−0.036	0.293	−0.011	0.826	−0.063	0.113
Experience × Prior healthcare job (no prior health related job)						
Manager	−0.004	0.530	−0.010	0.313	0.004	0.680
LPN/LVN	−0.009	0.001***	−0.024	0.003***	−0.003	0.345
Allied health	−0.003	0.338	−0.010	0.066*	0.003	0.375
Nursing aide	−0.006	0.015**	−0.009	0.01**	−0.002	0.501
Clerk	0.000	0.946	−0.004	0.572	0.005	0.425
Other	0.002	0.678	−0.001	0.886	0.006	0.194
Experience Square × Prior healthcare job (no prior health related job)						
Manager	−6.57E-05	0.736	1.23E-04	0.657	−3.12E-04	0.267
LPN/LVN	1.98E-04	0.015**	0.001	0.005***	5.57E-05	0.594
Allied health	8.54E-05	0.329	2.40E-04	0.082*	−6.55E-05	0.539
Nursing aide	1.24E-04	0.049**	1.85E-04	0.04**	4.93E-05	0.529
Clerk	−4.51E-05	0.701	4.70E-05	0.769	−1.61E-04	0.379
Other	6.89E-06	0.967	7.65E-05	0.781	−8.11E-05	0.575
Gender (Male)	−0.099	0.001***	−0.131	0.001***	−0.080	0.001***
Marital Status (Married)	−0.017	0.004***	−0.018	0.07*	−0.017	0.028**
Highest RN/RN-related education^c^						
Associate	Reference		NA		Reference	
Bachelor	0.044	0.001***	Reference		0.050	0.001***
Master's	0.247	0.001***	0.213	0.001***	0.232	0.001***
Medical, surgical and other specialists per 1,000 population	0.047	0.001***	0.042	0.001***	0.049	0.001***
Primary care practitioners per 1,000 population	−0.159	0.001***	−0.157	0.001***	−0.158	0.001***
Race - Other (white)	0.020	0.024**	0.030	0.009***	0.009	0.466
Work Status (Full-time no-overwork)						
Part-time	−0.077	0.001***	−0.085	0.001***	−0.072	0.001***
Full-time overwork	−0.121	0.001***	−0.128	0.001***	−0.117	0.001***
Work setting (Hospital)						
Nursing Home	−0.141	0.001***	−0.145	0.001***	−0.135	0.001***
Other setting	−0.130	0.001***	−0.141	0.001***	−0.124	0.001***
Urban	0.038	0.001***	0.023	0.102	0.043	0.001***
Constant	3.174	0.001***	3.222	0.001***	3.191	0.001***
Rho	−0.106	0.001***	−0.124	0.008***	−0.094	0.011**

*NA* not applicable, *LPN/LVN* Licensed Practical Nurses/Licensed Vocational Nurses

* *P* < 0.1, ** *P* < 0.05, *** *P* < 0.01

The additional covariates of 50 states (and Washington DC) in the regression model were not presented in this Table 2

‡ Logarithm form of Registered Nurse (RN) hourly wage; § Heckman’s Sample Selection model: The first-stage equation’s dependent variable was a dichotomous variable indicating working or not. The covariates uniquely included in the first-stage equation (i.e., excluded from the second-stage equation) were other household income, county-level characteristics (uninsurance rate and unemployment rate), age (five categories), student status (full-time, part-time, or no student) and children at home (four categories). Another set of covariates included in both the first-stage equation and the second-stage equation were six categories of prior healthcare employment (manager, LPN/LVN, allied health, nursing aide, clerk, and all other healthcare positions), race, gender, marital status, highest nursing degree, county-level characteristics (primary care practitioners per 1,000 population; and medical, surgical and other specialists per 1,000 population), indicators for 50 states (and Washington DC), and urban area. The second-stage equation’s estimates were presented in this Table 2. These Heckman models were justified by the statistically significant rho, reported in the bottom row of this Table 2; † NSSRN: nationally representative National Sample Survey of Registered Nurses;

^a^All actively licensed RNs in the NSSRN 2008 public use data file, excluding RNs who resided outside the United States and RNs whose initial nursing education was at the diploma or graduate (Master’s or Doctorate) level

^b^Experience was calculated by subtracting the year of first RN license from 2008. Additionally, one year was subtracted for RNs who left nursing for one or more years since becoming an RN (unweighted 11.7 % of sample), and 0.5 year was subtracted for RNs who are recent graduates and could not have left nursing for one or more years (unweighted 0.9 % of sample)

^c^Bachelor is the reference for the sample of Initial Bachelor degree only

The marginal effects reported in Table 2 were estimated at the mean of each variable in the multivariate regression models. We also estimated the marginal effects of years of RN experience and its squared term for every five years of experience (ranging from zero to 35 years) at the means of other variables. These estimates of the marginal effects were used to predict the effect of prior healthcare employment on wage growth over years of RNs experience for BSN-educated RNs (Figs. 1 and 2). Fig. 1 shows expected annual salary trajectories over RN experience among the four prior healthcare employment groups and the control group (without prior healthcare employment). Only these four prior healthcare employment groups were included in Fig. 1, since they were statistically different from the control group in either starting salary, experience-based wage growth or both. As explained earlier, at the starting point (i.e., when RN experience was zero), three prior healthcare employment groups had a higher annual salary compared to the control group (approximately $50,000 on average among the control group; shown in Fig. 1 but not presented in Tables) among BSN-educated nurses. The salary advantage of these three groups was $6500 for prior managers, $7700 for prior LPN/LVNs, and $3400 for prior nursing aides.

Among BSN-educated RNs, the annual salary trajectories over the RN experience differed noticeably across the groups in Fig. 1. For instance, the predicted annual salary of the prior manager group exceeds that of the control group throughout the entire RN career. This was because the prior manager group had a higher starting salary and equivalent wage growth with experience compared to the control group.

We also calculated the estimated difference in the lifetime earning of RNs (expressed as the area under the curve in Fig. 1), based on the results in Table 2. These calculations were not reported in tables for two reasons. First, the difference in lifetime earnings depends on the assumption about the discount rate, i.e., assigning a lower value for future earnings. For instance, without applying the discount rate, the control group without prior healthcare employment was estimated to earn $1,973,958 for working as RNs for 30 years, which was $24,000–$138,000 higher than other groups with prior employment as LPN/LVN, allied health or nursing aide among the BSN-educated RNs. Among these RNs, when the 3 % discounting rate is applied, the lifetime earnings of the control group declines to $1,279,035 (still in 2008 US dollars), which was $5900–$82,900 higher than other three groups. Second, these estimates were based on uncertain assumptions, e.g., that the current wage differences based on prior healthcare employment will persist for the next 30 years.

On the other hand, among those with prior LPN/LVN employment and prior nursing aide employment, their initial salary advantage over the control group disappeared once BSN-educated RN experience reached approximately ten years. This is because the predicted wage growth with experience was higher for the control group, as presented in Fig. 2. This advantage of the control group, in terms of the wage growth (i.e., financial return to RN experience), continues until the time when RN experience reaches 20 years. This advantage was observed as compared to RNs with prior LPN/LVN, allied health, and nursing aide.

Based on the findings in Table 2 and Figs. 1 and 2, our hypothesis 2 (RNs with prior healthcare employment will have different experience-based wage growth than the control group) was supported for BSN-educated RNs. Our hypothesis 3 (RNs with prior healthcare employment will have different starting wages and different experience-based wage growth, depending on the initial RN education, either BSN or Associate degree) was also supported, partly because the interaction terms between RN experience and prior healthcare employment were statistically significant only among BSN-educated RNs. This was also partly because the estimated coefficients of prior healthcare employment variables (measuring the starting wage difference) were different in magnitude and statistical significance between BSN-educated RNs and Associate-educated RNs.

## Discussion

This study indicated that prior healthcare employment was associated with a different starting RN wage and different wage growth over the period of RN experience, as hypothesized. These associations’ magnitudes vary, depending on the type of prior healthcare employment and the type of initial RN education. For instance, three categories of prior healthcare employment were associated with a higher starting wage among BSN-educated RNs, but only one category had a marginally significant (*p*-value = 0.096) association for Associate-educated RNs, thus supporting our hypothesis 1 particularly among BSN-educated RNs. Moreover, the magnitude of the association between the initial hourly wage and prior LPN/LVN employment was greater among BSN-educated RNs ($3.86) than Associate-educated RNs ($1.12). The higher wage return for RNs with a BSN, compared to RNs with an Associate degree, may motivate non-RN healthcare workers to seek a BSN in their transition to RN jobs, which could help advancement toward the 80 % BSN workforce recommended by the U.S. Institute of Medicine [7]. Additionally, our empirical results are important to develop policies to stabilize the RN workforce supply because RNs with prior healthcare employment have steadily grown in number and were found to be more likely to be employed, compared to RNs without prior healthcare employment [2].

Among BSN-educated RNs, a higher initial wage was found among three groups with prior healthcare employment: prior manager, prior LPN/LVN and prior nursing aide. There was statistically no difference in initial wage for other prior health care employment. Such variation across prior employment may be explained in two ways. Those with prior experience as managers, LPN/LVNs, or nursing aide may have specific work experience that is valued by employers. Prior healthcare employment does not appear to be valued by employers for Associate-educated RNs. This may be because prior employment does not increase the human capital of Associate-educated RNs, or it may be because Associate-educated RNs are less able to pursue employment that rewards prior employment. If Associate-educated RNs are less geographically mobile, for example, employers may be able to use monopsony power to constrain their wage.

On the other hand, among BSN-educated RNs, lower experience-based wage growth among three groups with prior healthcare employment might discourage those working as LPN/LVNs, allied health, and nursing aides from seeking a new RN career. Caution is needed to interpret these findings because experience-based wage growth also varied in its magnitude depending on the type of prior healthcare employment. For instance, the prior manager group among BSN-educated RNs had a higher starting salary and equivalent experience-based wage growth, compared to the control group (without any prior healthcare employment), which led to a higher annual salary for the prior manager group throughout their entire RN career. This may be because this group is more likely to hold a manger position that requires an RN license, and manager positions pay a higher salary than non-manager RN positions.

There are two potential reasons to explain why experience-based wage growth of RNs with prior healthcare employment was lower than that of the control group (without any prior healthcare employment) among BSN-educated RNs. One potential reason is that employers may have tried to achieve salary equity among all BSN-educated RNs by offsetting the higher starting wage among RNs with prior healthcare employment via lower experience-based wage growth. The second potential reason is the career path difference. Among BSN-educated RNs, RNs with prior employment as LPN/LVN, nursing aide, or allied health might not have a strong preference for working in hospitals that provide high human capital accumulation and higher wages [8, 16]. For instance, using the unadjusted proportion of work settings, BSN-educated RNs with prior employment as LPN/LVN were less likely to work in hospitals and more likely to work in nursing homes compared to the control group (*p* < 0.001). Similarly, RNs with prior employment as a nursing aide were less likely to work in hospitals than the control group (*p* < 0.001). However, this prior nursing aide group was less likely to work in nursing homes and more likely to work in other settings (e.g., nursing education, public health/community health, school health service, occupational health, ambulatory care, insurance claims/benefits, and others) compared to the control group, which did not obviously align with the second potential reason.

This study has a number of limitations. The validity of our primary analysis might be lowered by the limitations of the available data. First, due to the relatively small sample size of RNs with prior manager employment (unweighted *N* = 105/weighted *N* = 8809 among the working sample of BSN-educated RNs), the wage estimates of this group tend to be relatively less reliable than those for other larger groups. Second, the heterogeneity within each group of prior healthcare employment may have biased our regression estimates toward null. In other words, our estimates were likely to be conservative. Such heterogeneity partly stemmed from multiple prior healthcare employments, e.g., allied health and nursing aide, for one RN. We also ran another set of sensitivity analyses that included a larger number of groups corresponding to combinations of multiple prior healthcare employments. This set of analyses yielded unstable estimates due to small sample sizes of some groups. Third, since NSSRN data is a cross-sectional dataset without data on other potential wage determinants (such as change of institution, RNs being certified in a special organization, and unionization), our findings may change when accounting for these potential determinants in a longitudinal analysis. Fourth, since the NSSRN data does not include data about RNs who changed institutions or left nursing profession, our findings would be more useful for recruitment plans rather than retention plans. A future study analyzing a large dataset, e.g., NSSRN, linked with detailed retention history and potential retention determinants (such as job satisfaction, professional autonomy, intellectual stimulation, excess workload and external market factors [17–19]) would make a contribution to the literature.

Also, future studies need to examine if wage growth differences reflect potential differences in career paths (e.g., work setting) or human capital (e.g., productivity). These studies could assess whether the current wage growth difference is justifiable or needs to be changed to prevent non-RN healthcare workers from being discouraged in seeking a new RN career. In addition, to examine the potential global wage effect on incentives to become an RN, a future analysis is expected to include both healthcare workers who became RNs and those who did not, comparing the wage differences among non-RN health related occupations, RNs and alternative non-health-related occupations.

## Conclusions

Prior employment in health-related jobs appears to increase the starting salary but reduce experience-based wage growth compared to RNs who do not have prior health-related employment, particularly for BSN-educated RNs. The higher wage return for BSN-education may motivate non-RN healthcare workers to seek a BSN (rather than an Associate degree) in their transition to RN jobs, which could help advancement toward the 80 % BSN workforce recommended by the U.S. Institute of Medicine.

## Additional files

Additional file 1: Table S1. A set of sensitivity analyses for Table 2, not mentioned in the main text. Compared Table 2, the models for Table S1 included the additional covariates indicating years-of-experience after obtaining Master's or Doctoral degree in nursing. (DOCX 53 kb) Additional file 2: Table S2. A set of sensitivity analyses for Table 2, not mentioned in the main text. Unlike Table 2 (including a continuous variable of “years-of-experience” as RN), the models for Table S2 included a set of dichotomous variables representing 1-year interval for experience as RN. (DOCX 103 kb) Additional file 3: Table S3. A set of sensitivity analyses for Table 2, not mentioned in the main text. Unlike Table 2 (including a continuous variable of “years-of-experience” as RNs), the models for Table S3 included a set of dichotomous variables indicating 4-year interval for experience as RN. (DOCX 56 kb) Additional file 4: Table S4. Supplemental analyses for Table 1, comparing demographic characteristics between “All sample” and “Working sample,” regarding the number of observations for each covariate. Table S4 consists of six tables below. Table S4-1: Unweighted Total Population (combining Initial Associate and BSN degree). Table S4-2: Unweighted Population (Initial Bachelor degree). Table S4-3: Unweighted Population (Initial Associate degree). Table S4-4: Weighted Total Population (combining Initial Associate and BSN degree). Table S4-5: Weighted Population (Initial Bachelor degree). Table S4-6: Weighted Population (Initial Associate degree). (DOCX 88 kb)

## Abbreviations

AHRF: Area Health Resource File
BSN: Bachelor of Science in Nursing
LPN/LVN: Licensed Practical Nurses or Licensed Vocational Nurses
NSSRN: National Sample Survey of Registered Nurses
RN: Registered Nurse
