# Supplementary material for: The effect of prior healthcare employment on the wages of registered nurses
Source: BMC Health Serv Res. 2016 Aug 19;16:412. doi: 10.1186/s12913-016-1667-0 (PMC4992246; doi:10.1186/s12913-016-1667-0)
Supplement: Additional file 1: Table S1. — A set of sensitivity analyses for Table 2, not mentioned in the main text. Compared Table 2, the models for Table S1 included the additional covariates indicating years-of-experience after obtaining Master's or Doctoral degree in nursing. (DOCX 53 kb) [file 12913_2016_1667_MOESM1_ESM.docx]

**Table S1. Determinants of RN hourly wage‡ based on Heckman model§ in 2008 NSSRN† data (Compared Table 2, the models for Table S1 included the additional covariates indicating years-of-experience after obtaining Master’s or Doctoral (MS/DR) degree in nursing)**

| Dependent variable: Log of RN Hourly Wage | | Total Population^1^  Weighted N=2,903,177  Un-weighted N=31,385 | | Initial Bachelor degree  Weighted N=967,075  Un-weighted N=10,565 | | Initial Associate degree  Weighted N=1,320,130  Un-weighted N=14,057 | | Initial Associate and Bachelor degree  Weighted N=2,287,205  Un-weighted N=24,622 | |
| --- | --- | --- | --- | --- | --- | --- | --- | --- | --- |
| Explanatory variable (Reference category) |  | Coefficient | P>\|t\| | Coefficient | P>\|t\| | Coefficient | P>\|t\| | Coefficient | P>\|t\| |
| MS/DR Experience^2^ |  | 0.001 | 0.774 | -0.006 | 0.105 | 0.009 | 0.164 | -0.001 | 0.782 |
| MS/DR Experience Square |  | -8.03E-05 | 0.311 | 1.27E-04 | 0.263 | -2.35E-04 | 0.130 | 1.88E-06 | 0.984 |
| MS/DR Experience × Prior healthcare job (no prior health related job) |  |  |  |  |  |  |  |  |  |
|  | Manager | -0.005 | 0.672 | -0.013 | 0.394 | -0.012 | 0.681 | -0.018 | 0.093 |
|  | LPN | 0.003 | 0.617 | 0.008 | 0.343 | 0.005 | 0.648 | 0.005 | 0.482 |
|  | Allied health | 0.004 | 0.368 | 0.005 | 0.568 | -0.009 | 0.214 | -0.002 | 0.669 |
|  | Nursing aide | 0.001 | 0.811 | 0.005 | 0.222 | 0.001 | 0.839 | 0.003 | 0.335 |
|  | Clerk | -0.002 | 0.815 | -0.012 | 0.128 | -0.012 | 0.594 | -0.011 | 0.142 |
|  | Other | 0.005 | 0.549 | 0.001 | 0.923 | 0.005 | 0.786 | 0.002 | 0.838 |
| MS/DR Experience Square × Prior healthcare job (no prior health related job) |  |  |  |  |  |  |  |  |  |
|  | Manager | 2.68E-04 | 0.629 | 0.001 | 0.245 | -2.51E-04 | 0.909 | 0.001 | 0.101 |
|  | LPN | -1.87E-04 | 0.439 | -3.68E-04 | 0.257 | -3.00E-04 | 0.499 | -2.87E-04 | 0.325 |
|  | Allied health | -1.21E-04 | 0.511 | -5.00E-05 | 0.887 | 1.51E-04 | 0.568 | 8.81E-05 | 0.706 |
|  | Nursing aide | -4.97E-05 | 0.589 | -2.30E-04 | 0.097 | -2.14E-04 | 0.293 | -1.96E-04 | 0.088 |
|  | Clerk | 1.33E-04 | 0.586 | 3.60E-04 | 0.127 | 4.63E-04 | 0.600 | 3.46E-04 | 0.153 |
|  | Other | -1.38E-04 | 0.619 | 4.51E-05 | 0.914 | -3.49E-04 | 0.622 | -4.95E-05 | 0.885 |
| Experience^3^ |  | 0.018 | 0.000 | 0.027 | 0.000 | 0.017 | 0.000 | 0.022 | 0.000 |
| Experience Square |  | -2.96E-04 | 0.000 | -0.001 | 0.000 | -3.29E-04 | 0.000 | -4.39E-04 | 0.000 |
| Prior healthcare job (no prior healthcare job) |  |  |  |  |  |  |  |  |  |
| Manager |  | 0.062 | 0.094 | 0.156 | 0.026 | 0.035 | 0.513 | 0.081 | 0.058 |
| LPN |  | 0.057 | 0.004 | 0.116 | 0.070 | 0.036 | 0.151 | 0.072 | 0.001 |
| Allied health |  | -0.019 | 0.342 | 0.054 | 0.136 | -0.023 | 0.392 | 0.013 | 0.533 |
| Nursing aide |  | 0.018 | 0.272 | 0.067 | 0.006 | 0.010 | 0.690 | 0.041 | 0.026 |
| Clerk |  | -1.49E-04 | 0.996 | -0.003 | 0.948 | -0.029 | 0.491 | -0.008 | 0.793 |
| Other |  | -0.030 | 0.369 | 0.033 | 0.534 | -0.043 | 0.282 | -0.006 | 0.876 |
| Experience × Prior healthcare job (no prior health related job) |  |  |  |  |  |  |  |  |  |
|  | Manager | 0.003 | 0.508 | -0.008 | 0.487 | 0.008 | 0.342 | 0.002 | 0.768 |
|  | LPN | -0.007 | 0.008 | -0.022 | 0.003 | -0.004 | 0.300 | -0.010 | 0.001 |
|  | Allied health | 0.002 | 0.407 | -0.011 | 0.042 | 0.002 | 0.509 | -0.003 | 0.270 |
|  | Nursing aide | -0.003 | 0.147 | -0.011 | 0.002 | -0.002 | 0.472 | -0.007 | 0.007 |
|  | Clerk | 0.001 | 0.808 | -4.15E-04 | 0.946 | 0.007 | 0.243 | 0.003 | 0.519 |
|  | Other | 0.003 | 0.568 | -0.007 | 0.500 | 0.002 | 0.632 | -0.002 | 0.748 |
| Experience Square × Prior healthcare job (no prior health related job) |  |  |  |  |  |  |  |  |  |
|  | Manager | -2.59E-04 | 0.089 | -6.49E-05 | 0.837 | -4.41E-04 | 0.127 | -2.83E-04 | 0.182 |
|  | LPN | 1.41E-04 | 0.043 | 0.001 | 0.004 | 7.92E-05 | 0.437 | 2.28E-04 | 0.006 |
|  | Allied health | -7.79E-05 | 0.282 | 2.41E-04 | 0.089 | -2.70E-05 | 0.806 | 9.26E-05 | 0.299 |
|  | Nursing aide | 4.85E-05 | 0.262 | 2.62E-04 | 0.007 | 7.11E-05 | 0.380 | 1.72E-04 | 0.011 |
|  | Clerk | -4.35E-05 | 0.756 | -6.29E-06 | 0.970 | -2.22E-04 | 0.221 | -1.01E-04 | 0.404 |
|  | Other | -3.44E-05 | 0.779 | 1.85E-04 | 0.564 | 4.00E-05 | 0.797 | 1.01E-04 | 0.596 |
| Gender (Male) |  | -0.107 | 0.000 | -0.132 | 0.000 | -0.083 | 0.000 | -0.102 | 0.000 |
| Marital Status (Married) |  | -0.017 | 0.002 | -0.019 | 0.050 | -0.015 | 0.054 | -0.017 | 0.005 |
| Highest RN/RN-related education^4^ |  |  |  |  |  |  |  |  |  |
| Diploma |  | 0.008 | 0.324 | (omitted) |  | (omitted) |  | (omitted) |  |
| Associate |  | Reference |  | (omitted) |  | Reference |  | Reference |  |
| Bachelor |  | 0.047 | 0.000 | Reference |  | 0.049 | 0.000 | 0.044 | 0.000 |
| Master’s |  | 0.249 | 0.000 | 0.253 | 0.000 | 0.205 | 0.000 | 0.266 | 0.000 |
| Medical, surgical and other specialists per 1,000 population |  | 0.057 | 0.000 | 0.054 | 0.000 | 0.062 | 0.000 | 0.059 | 0.000 |
| Primary care practitioners per 1,000 population |  | -0.218 | 0.000 | -0.228 | 0.000 | -0.220 | 0.000 | -0.222 | 0.000 |
| Race - Other (white) |  | 0.031 | 0.000 | 0.047 | 0.000 | 0.023 | 0.044 | 0.037 | 0.000 |
| Region of employment or residence (New England) |  |  |  |  |  |  |  |  |  |
| Middle Atlantic |  | -0.040 | 0.000 | -0.001 | 0.930 | -0.065 | 0.000 | -0.037 | 0.002 |
| East North Central |  | -0.108 | 0.000 | -0.098 | 0.000 | -0.112 | 0.000 | -0.104 | 0.000 |
| West North Central |  | -0.127 | 0.000 | -0.095 | 0.000 | -0.134 | 0.000 | -0.116 | 0.000 |
| South Atlantic |  | -0.089 | 0.000 | -0.062 | 0.000 | -0.110 | 0.000 | -0.090 | 0.000 |
| East South Central |  | -0.163 | 0.000 | -0.149 | 0.000 | -0.181 | 0.000 | -0.167 | 0.000 |
| West South Central |  | -0.071 | 0.000 | -0.059 | 0.005 | -0.084 | 0.000 | -0.072 | 0.000 |
| Mountain |  | -0.026 | 0.020 | 0.006 | 0.747 | -0.045 | 0.006 | -0.023 | 0.076 |
| Pacific |  | 0.160 | 0.000 | 0.163 | 0.000 | 0.160 | 0.000 | 0.162 | 0.000 |
| Work Status (Full-time no-overwork) |  |  |  |  |  |  |  |  |  |
| Part-time |  | -0.073 | 0.000 | -0.078 | 0.000 | -0.062 | 0.000 | -0.069 | 0.000 |
| Full-time overwork |  | -0.120 | 0.000 | -0.128 | 0.000 | -0.116 | 0.000 | -0.120 | 0.000 |
| Work setting (Hospital) |  |  |  |  |  |  |  |  |  |
| Nursing Home |  | -0.147 | 0.000 | -0.151 | 0.000 | -0.136 | 0.000 | -0.142 | 0.000 |
| Other setting |  | -0.137 | 0.000 | -0.140 | 0.000 | -0.121 | 0.000 | -0.129 | 0.000 |
| Constant |  | 3.419 | 0.000 | 3.424 | 0.000 | 3.407 | 0.000 | 3.385 | 0.000 |

Note: * P<0.1, ** P<0.05, *** P<0.01; NA: not applicable; LPN/LVN: Licensed Practical Nurses/Licensed Vocational Nurses

‡ Logarithm form of Registered Nurse (RN) hourly wage; § Heckman’s Sample Selection model: The first-stage equation’s dependent variable was a dichotomous variable indicating working or not. The covariates uniquely included in the first-stage equation (i.e., excluded from the second-stage equation) were other household income, county-level characteristics (uninsurance rate and unemployment rate), age (five categories), student status (full-time, part-time, or no student) and children at home (four categories). Another set of covariates included in both the first-stage equation and the second-stage equation were six categories of prior healthcare employment (manager, LPN/LVN, allied health, nursing aide, clerk, and all other healthcare positions), race, gender, marital status, highest nursing degree, county-level characteristics (primary care practitioners per 1,000 population; and medical, surgical and other specialists per 1,000 population), and indicators for 9 census regions. The second-stage equation’s estimates were presented in this Table S1.; † NSSRN: nationally representative National Sample Survey of Registered Nurses;

^1^ All actively licensed RNs in the NSSRN 2008 public use data file, excluding RNs who resided outside the United States only (i.e., the same as the total population for the first submission). Thus, this “old” total population still included RNs whose initial nursing education was at the diploma or graduate (Master’s or Doctorate) level.

^2^ This variable indicates the years after a subject obtained a highest graduated degree in nursing. It was created to estimate the unique effects of “year-of-experience and prior healthcare jobs” among RNs with Master’s or Doctoral degree in nursing, in addition to another “year of experience (after becoming RN)” variable (among all RNs) detailed in the footnote (3) below. This variable was created from the NSSRN data including the specific year when these highest degrees in nursing were obtained.

^3^ Experience was calculated by subtracting the year of first RN license from 2008. Additionally, one year was subtracted for RNs who left nursing for one or more years since becoming an RN (unweighted 11.7% of sample), and 0.5 year was subtracted for RNs who are recent graduates and could not have left nursing for one or more years (unweighted 0.9% of sample).

^4^ Bachelor is the reference for the sample of Initial Bachelor degree only.
