# Supplementary material for: The effect of prior healthcare employment on the wages of registered nurses
Source: BMC Health Serv Res. 2016 Aug 19;16:412. doi: 10.1186/s12913-016-1667-0 (PMC4992246; doi:10.1186/s12913-016-1667-0)
Supplement: Additional file 2: Table S2. — A set of sensitivity analyses for Table 2, not mentioned in the main text. Unlike Table 2 (including a continuous variable of “years-of-experience” as RN), the models for Table S2 included a set of dichotomous variables representing 1-year interval for experience as RN. (DOCX 103 kb) [file 12913_2016_1667_MOESM2_ESM.docx]

**Table S2. Determinants of RN hourly wage‡ based on Heckman model§ in 2008 NSSRN† data (including a set of dichotomous variables representing 1-year interval for experience as RN)**

| Dependent variable: Log of RN Hourly Wage | | Total Population^1^  Weighted N=2,903,220  Un-weighted N=31,386 | | Initial Bachelor degree  Weighted N=967,075  Un-weighted N=10,565 | | Initial Associate degree  Weighted N=1,320,130  Un-weighted N=14,057 | |  |
| --- | --- | --- | --- | --- | --- | --- | --- | --- |
| Explanatory variable (Reference category) | | Coefficient | P>\|t\| | Coefficient | P>\|t\| | Coefficient | P>\|t\| | |
| Prior healthcare job (no prior healthcare job) | |  |  |  |  |  |  | |
| Manager |  | 0.167 | 0.039 | 0.278 | 0.000 | 0.078 | 0.361 | |
| LPN |  | 0.097 | 0.167 | -0.170 | 0.163 | 0.051 | 0.416 | |
| Allied health |  | 0.034 | 0.630 | 0.190 | 0.077 | -0.101 | 0.328 | |
| Nursing aide |  | 0.031 | 0.513 | 0.062 | 0.356 | -0.025 | 0.641 | |
| Clerk |  | 0.092 | 0.416 | -0.054 | 0.492 | 0.118 | 0.378 | |
| Other |  | 0.111 | 0.091 | 0.020 | 0.855 | 0.084 | 0.127 | |
| Experience (0 Year)^2^ |  |  |  |  |  |  |  | |
| Year 1 |  | 0.065 | 0.144 | 0.157 | 0.021 | -0.054 | 0.347 | |
| Year 2 |  | 0.148 | 0.000 | 0.169 | 0.016 | 0.109 | 0.027 | |
| Year 3 |  | 0.237 | 0.000 | 0.316 | 0.000 | 0.127 | 0.018 | |
| Year 4 |  | 0.222 | 0.000 | 0.289 | 0.000 | 0.124 | 0.004 | |
| Year 5 |  | 0.226 | 0.000 | 0.311 | 0.000 | 0.124 | 0.022 | |
| Year 6 |  | 0.218 | 0.000 | 0.287 | 0.000 | 0.126 | 0.049 | |
| Year 7 |  | 0.336 | 0.000 | 0.412 | 0.000 | 0.270 | 0.000 | |
| Year 8 |  | 0.306 | 0.000 | 0.320 | 0.001 | 0.264 | 0.000 | |
| Year 9 |  | 0.325 | 0.000 | 0.298 | 0.000 | 0.254 | 0.000 | |
| Year 10 |  | 0.332 | 0.000 | 0.419 | 0.000 | 0.217 | 0.000 | |
| Year 11 |  | 0.355 | 0.000 | 0.405 | 0.000 | 0.274 | 0.000 | |
| Year 12 |  | 0.366 | 0.000 | 0.474 | 0.000 | 0.250 | 0.000 | |
| Year 13 |  | 0.315 | 0.000 | 0.409 | 0.000 | 0.203 | 0.000 | |
| Year 14 |  | 0.361 | 0.000 | 0.433 | 0.000 | 0.235 | 0.000 | |
| Year 15 |  | 0.400 | 0.000 | 0.462 | 0.000 | 0.319 | 0.000 | |
| Year 16 |  | 0.352 | 0.000 | 0.457 | 0.000 | 0.232 | 0.000 | |
| Year 17 |  | 0.380 | 0.000 | 0.442 | 0.000 | 0.266 | 0.000 | |
| Year 18 |  | 0.332 | 0.000 | 0.445 | 0.000 | 0.161 | 0.001 | |
| Year 19 |  | 0.409 | 0.000 | 0.456 | 0.000 | 0.282 | 0.000 | |
| Year 20 |  | 0.383 | 0.000 | 0.506 | 0.000 | 0.211 | 0.001 | |
| Year 21 |  | 0.365 | 0.000 | 0.434 | 0.000 | 0.280 | 0.000 | |
| Year 22 |  | 0.393 | 0.000 | 0.476 | 0.000 | 0.265 | 0.000 | |
| Year 23 |  | 0.389 | 0.000 | 0.497 | 0.000 | 0.262 | 0.000 | |
| Year 24 |  | 0.370 | 0.000 | 0.435 | 0.000 | 0.322 | 0.000 | |
| Year 25 |  | 0.399 | 0.000 | 0.490 | 0.000 | 0.296 | 0.000 | |
| Year 26 |  | 0.329 | 0.000 | 0.429 | 0.000 | 0.174 | 0.031 | |
| Year 27 |  | 0.400 | 0.000 | 0.514 | 0.000 | 0.294 | 0.000 | |
| Year 28 |  | 0.361 | 0.000 | 0.470 | 0.000 | 0.175 | 0.018 | |
| Year 29 |  | 0.361 | 0.000 | 0.504 | 0.000 | 0.240 | 0.000 | |
| Year 30 |  | 0.386 | 0.000 | 0.435 | 0.000 | 0.292 | 0.001 | |
| Year 31 |  | 0.466 | 0.000 | 0.512 | 0.000 | 0.365 | 0.000 | |
| Year 32 |  | 0.396 | 0.000 | 0.542 | 0.000 | 0.252 | 0.000 | |
| Year 33 |  | 0.409 | 0.000 | 0.474 | 0.000 | 0.438 | 0.000 | |
| Year 34 |  | 0.319 | 0.000 | 0.467 | 0.000 | 0.217 | 0.003 | |
| Year 35 |  | 0.369 | 0.000 | 0.422 | 0.000 | 0.252 | 0.001 | |
| Year 36 |  | 0.341 | 0.000 | 0.312 | 0.001 | 0.327 | 0.000 | |
| Year 37 |  | 0.311 | 0.000 | 0.345 | 0.000 | 0.180 | 0.002 | |
| Year 38 |  | 0.403 | 0.000 | 0.554 | 0.000 | 0.398 | 0.000 | |
| Year 39 |  | 0.315 | 0.000 | 0.293 | 0.002 | 0.238 | 0.000 | |
| Year 40 |  | 0.412 | 0.000 | 0.400 | 0.000 | 0.288 | 0.000 | |
| Year 41 |  | 0.406 | 0.000 | 0.357 | 0.039 | 0.259 | 0.000 | |
| Year 42 |  | 0.399 | 0.000 | 0.328 | 0.127 | 0.277 | 0.000 | |
| Year >=43 |  | 0.329 | 0.000 | 0.329 | 0.001 | 0.116 | 0.278 | |
| Interaction terms |  |  |  |  |  |  |  | |
| Manager | Year 1 | 0.013 | 0.907 | -0.058 | 0.475 | 0.134 | 0.357 | |
|  | Year 2 | -0.097 | 0.378 | -0.105 | 0.777 | -0.076 | 0.488 | |
|  | Year 3 | -0.178 | 0.101 | -0.405 | 0.000 | -0.043 | 0.733 | |
|  | Year 4 | -0.142 | 0.154 | -0.185 | 0.116 | -0.107 | 0.319 | |
|  | Year 5 | -0.054 | 0.536 | -0.194 | 0.033 | 0.061 | 0.538 | |
|  | Year 6 | 0.100 | 0.604 | 0.179 | 0.686 | -0.014 | 0.899 | |
|  | Year 7 | -0.121 | 0.344 | -0.105 | 0.209 | -0.149 | 0.377 | |
|  | Year 8 | -0.122 | 0.324 | -0.354 | 0.163 | -0.049 | 0.723 | |
|  | Year 9 | 0.018 | 0.884 | -0.002 | 0.975 | 0.074 | 0.529 | |
|  | Year 10 | -0.189 | 0.060 | -0.285 | 0.027 | -0.016 | 0.868 | |
|  | Year 11 | -0.182 | 0.094 | -0.235 | 0.003 | -0.118 | 0.374 | |
|  | Year 12 | -0.187 | 0.097 | -0.568 | 0.000 | 0.034 | 0.809 | |
|  | Year 13 | -0.131 | 0.188 | -0.177 | 0.086 | -0.036 | 0.757 | |
|  | Year 14 | -0.248 | 0.102 | (omitted) |  | 0.009 | 0.944 | |
|  | Year 15 | -0.284 | 0.006 | -0.407 | 0.000 | -0.179 | 0.101 | |
|  | Year 16 | -0.203 | 0.058 | -0.266 | 0.024 | -0.119 | 0.330 | |
|  | Year 17 | -0.151 | 0.146 | -0.202 | 0.152 | -0.080 | 0.537 | |
|  | Year 18 | -0.100 | 0.484 | -0.048 | 0.773 | -0.048 | 0.737 | |
|  | Year 19 | -0.261 | 0.102 | -0.104 | 0.503 | -0.278 | 0.010 | |
|  | Year 20 | -0.094 | 0.328 | -0.239 | 0.026 | 0.039 | 0.755 | |
|  | Year 21 | -0.083 | 0.597 | -0.322 | 0.000 | -0.221 | 0.029 | |
|  | Year 22 | -0.625 | 0.000 | -0.721 | 0.000 | -0.507 | 0.000 | |
|  | Year 23 | 0.155 | 0.106 | 0.052 | 0.477 | 0.272 | 0.019 | |
|  | Year 24 | -0.197 | 0.146 | -0.314 | 0.103 | -0.166 | 0.150 | |
|  | Year 25 | -0.048 | 0.731 | -0.160 | 0.048 | 0.046 | 0.731 | |
|  | Year 26 | -0.189 | 0.044 | -0.313 | 0.002 | -0.042 | 0.717 | |
|  | Year 27 | -0.188 | 0.266 | -0.169 | 0.019 | -0.180 | 0.628 | |
|  | Year 28 | 0.018 | 0.912 | -0.371 | 0.424 | -0.074 | 0.605 | |
|  | Year 29 | -0.504 | 0.000 | -0.718 | 0.000 | (omitted) |  | |
|  | Year 30 | -0.352 | 0.126 | -1.258 | 0.000 | -0.041 | 0.851 | |
|  | Year 31 | -0.257 | 0.148 | (omitted) |  | -0.076 | 0.469 | |
|  | Year 32 | -0.339 | 0.017 | -0.336 | 0.000 | -0.297 | 0.095 | |
|  | Year 33 | -0.310 | 0.111 | -0.171 | 0.032 | -0.363 | 0.083 | |
|  | Year 34 | 0.498 | 0.000 | 0.384 | 0.000 | 0.011 | 0.918 | |
|  | Year 35 | (omitted) |  | (omitted) |  | (omitted) |  | |
|  | Year 36 | -0.320 | 0.000 | (omitted) |  | (omitted) |  | |
|  | Year 37 | -0.699 | 0.110 | -0.646 | 0.186 | -1.198 | 0.000 | |
|  | Year 38 | -0.043 | 0.635 | (omitted) |  | -0.061 | 0.545 | |
|  | Year 39 | (omitted) |  | (omitted) |  | (omitted) |  | |
|  | Year 40 | -0.356 | 0.000 | (omitted) |  | (omitted) |  | |
|  | Year 41 | -0.123 | 0.168 | -0.066 | 0.698 | (omitted) |  | |
|  | Year 42 | (omitted) |  | (omitted) |  | (omitted) |  | |
|  | Year >=43 | -0.601 | 0.000 | -0.677 | 0.000 | (omitted) |  | |
| LPN | Year 1 | -0.033 | 0.664 | 0.254 | 0.085 | 0.030 | 0.689 | |
|  | Year 2 | 0.012 | 0.876 | 0.461 | 0.001 | 0.001 | 0.987 | |
|  | Year 3 | -0.059 | 0.482 | 0.311 | 0.043 | -0.016 | 0.850 | |
|  | Year 4 | -0.146 | 0.062 | 0.137 | 0.316 | -0.108 | 0.132 | |
|  | Year 5 | -0.021 | 0.790 | 0.163 | 0.285 | 0.037 | 0.646 | |
|  | Year 6 | 0.021 | 0.792 | 0.363 | 0.025 | 0.065 | 0.422 | |
|  | Year 7 | -0.128 | 0.123 | 0.011 | 0.946 | -0.101 | 0.283 | |
|  | Year 8 | -0.078 | 0.383 | 0.207 | 0.197 | -0.069 | 0.453 | |
|  | Year 9 | -0.168 | 0.042 | -0.051 | 0.742 | -0.090 | 0.319 | |
|  | Year 10 | -0.126 | 0.131 | 0.036 | 0.829 | -0.050 | 0.526 | |
|  | Year 11 | -0.112 | 0.145 | 0.147 | 0.324 | -0.079 | 0.284 | |
|  | Year 12 | -0.123 | 0.149 | 0.135 | 0.400 | -0.071 | 0.391 | |
|  | Year 13 | -0.118 | 0.165 | 0.119 | 0.428 | -0.050 | 0.570 | |
|  | Year 14 | -0.161 | 0.046 | 0.310 | 0.127 | -0.114 | 0.112 | |
|  | Year 15 | -0.158 | 0.055 | 0.066 | 0.628 | -0.121 | 0.121 | |
|  | Year 16 | -0.154 | 0.062 | -0.063 | 0.721 | -0.049 | 0.506 | |
|  | Year 17 | -0.188 | 0.018 | 0.092 | 0.496 | -0.123 | 0.101 | |
|  | Year 18 | -0.102 | 0.283 | 0.016 | 0.913 | 0.041 | 0.643 | |
|  | Year 19 | -0.202 | 0.015 | 0.012 | 0.959 | -0.104 | 0.239 | |
|  | Year 20 | -0.077 | 0.342 | 0.046 | 0.791 | 0.064 | 0.381 | |
|  | Year 21 | -0.091 | 0.270 | 0.165 | 0.265 | -0.040 | 0.615 | |
|  | Year 22 | -0.117 | 0.121 | 0.024 | 0.870 | -0.017 | 0.809 | |
|  | Year 23 | -0.164 | 0.047 | -0.004 | 0.979 | -0.085 | 0.289 | |
|  | Year 24 | -0.051 | 0.549 | 0.154 | 0.255 | -0.041 | 0.630 | |
|  | Year 25 | -0.161 | 0.044 | 0.082 | 0.557 | -0.142 | 0.145 | |
|  | Year 26 | -0.012 | 0.891 | 0.221 | 0.232 | 0.095 | 0.381 | |
|  | Year 27 | -0.175 | 0.152 | -0.153 | 0.347 | -0.114 | 0.407 | |
|  | Year 28 | -0.031 | 0.690 | 0.130 | 0.354 | 0.138 | 0.102 | |
|  | Year 29 | -0.021 | 0.817 | 0.195 | 0.190 | 0.064 | 0.516 | |
|  | Year 30 | -0.115 | 0.260 | 0.230 | 0.182 | -0.147 | 0.265 | |
|  | Year 31 | -0.147 | 0.251 | 0.211 | 0.128 | -0.151 | 0.419 | |
|  | Year 32 | -0.068 | 0.483 | -0.009 | 0.971 | 0.085 | 0.442 | |
|  | Year 33 | -0.129 | 0.145 | 0.112 | 0.375 | -0.246 | 0.021 | |
|  | Year 34 | -0.007 | 0.930 | 0.192 | 0.119 | 0.028 | 0.812 | |
|  | Year 35 | -0.203 | 0.034 | 0.125 | 0.449 | -0.156 | 0.179 | |
|  | Year 36 | -0.072 | 0.664 | 0.163 | 0.285 | -0.339 | 0.091 | |
|  | Year 37 | 0.140 | 0.285 | 0.676 | 0.172 | 0.306 | 0.002 | |
|  | Year 38 | -0.092 | 0.434 | 0.154 | 0.381 | -0.127 | 0.311 | |
|  | Year 39 | -0.174 | 0.188 | 0.040 | 0.787 | -0.105 | 0.518 | |
|  | Year 40 | -0.269 | 0.001 | (omitted) |  | -0.215 | 0.015 | |
|  | Year 41 | 0.203 | 0.155 | -0.022 | 0.912 | 0.370 | 0.017 | |
|  | Year 42 | -0.299 | 0.080 | 0.295 | 0.582 | -0.532 | 0.000 | |
|  | Year >=43 | 0.128 | 0.347 | 0.099 | 0.487 | (omitted) |  | |
| Allied health | Year 1 | 0.006 | 0.943 | -0.226 | 0.057 | 0.214 | 0.074 | |
|  | Year 2 | -0.033 | 0.666 | -0.179 | 0.140 | 0.068 | 0.558 | |
|  | Year 3 | -0.090 | 0.246 | -0.158 | 0.194 | 0.017 | 0.884 | |
|  | Year 4 | -0.056 | 0.473 | -0.198 | 0.084 | 0.104 | 0.367 | |
|  | Year 5 | -0.037 | 0.659 | -0.160 | 0.285 | 0.079 | 0.471 | |
|  | Year 6 | -0.003 | 0.967 | -0.197 | 0.080 | 0.130 | 0.290 | |
|  | Year 7 | -0.131 | 0.123 | -0.266 | 0.033 | -0.036 | 0.766 | |
|  | Year 8 | -0.074 | 0.348 | -0.187 | 0.159 | 0.024 | 0.855 | |
|  | Year 9 | -0.049 | 0.594 | -0.120 | 0.341 | 0.080 | 0.559 | |
|  | Year 10 | -0.052 | 0.548 | -0.284 | 0.034 | 0.130 | 0.289 | |
|  | Year 11 | 0.004 | 0.965 | -0.169 | 0.176 | 0.155 | 0.193 | |
|  | Year 12 | -0.110 | 0.196 | -0.285 | 0.027 | 0.018 | 0.882 | |
|  | Year 13 | 0.017 | 0.839 | -0.145 | 0.249 | 0.206 | 0.088 | |
|  | Year 14 | -0.039 | 0.621 | -0.181 | 0.200 | 0.115 | 0.310 | |
|  | Year 15 | -0.063 | 0.411 | -0.282 | 0.021 | 0.073 | 0.495 | |
|  | Year 16 | -0.029 | 0.731 | -0.246 | 0.091 | 0.145 | 0.254 | |
|  | Year 17 | -0.094 | 0.315 | -0.133 | 0.316 | 0.016 | 0.903 | |
|  | Year 18 | 0.079 | 0.385 | 0.004 | 0.977 | 0.202 | 0.082 | |
|  | Year 19 | -0.072 | 0.414 | -0.226 | 0.120 | 0.102 | 0.439 | |
|  | Year 20 | -0.109 | 0.227 | -0.531 | 0.001 | 0.188 | 0.064 | |
|  | Year 21 | -0.123 | 0.132 | -0.277 | 0.052 | -0.016 | 0.905 | |
|  | Year 22 | -0.080 | 0.366 | -0.174 | 0.246 | 0.108 | 0.326 | |
|  | Year 23 | -0.109 | 0.171 | -0.295 | 0.025 | 0.071 | 0.542 | |
|  | Year 24 | -0.045 | 0.591 | -0.187 | 0.161 | 0.015 | 0.897 | |
|  | Year 25 | -0.085 | 0.359 | -0.285 | 0.061 | 0.028 | 0.834 | |
|  | Year 26 | 0.025 | 0.774 | -0.237 | 0.091 | 0.238 | 0.092 | |
|  | Year 27 | -0.011 | 0.915 | -0.189 | 0.115 | 0.139 | 0.387 | |
|  | Year 28 | -0.137 | 0.280 | -0.288 | 0.433 | 0.081 | 0.612 | |
|  | Year 29 | -0.036 | 0.658 | -0.231 | 0.079 | 0.066 | 0.593 | |
|  | Year 30 | 0.040 | 0.685 | -0.327 | 0.071 | 0.258 | 0.071 | |
|  | Year 31 | -0.011 | 0.904 | -0.197 | 0.152 | 0.090 | 0.523 | |
|  | Year 32 | -0.115 | 0.311 | -0.234 | 0.338 | -0.049 | 0.784 | |
|  | Year 33 | 0.032 | 0.773 | 0.095 | 0.724 | 0.003 | 0.987 | |
|  | Year 34 | 0.193 | 0.178 | -0.064 | 0.635 | 0.506 | 0.040 | |
|  | Year 35 | -0.063 | 0.551 | -0.276 | 0.041 | 0.249 | 0.082 | |
|  | Year 36 | 0.087 | 0.638 | (omitted) |  | 0.262 | 0.175 | |
|  | Year 37 | 0.110 | 0.422 | 0.089 | 0.800 | 0.022 | 0.889 | |
|  | Year 38 | -0.053 | 0.615 | -0.594 | 0.011 | 0.030 | 0.807 | |
|  | Year 39 | 0.208 | 0.157 | 0.267 | 0.058 | (omitted) |  | |
|  | Year 40 | -0.028 | 0.772 | -0.171 | 0.435 | 0.156 | 0.169 | |
|  | Year 41 | -0.094 | 0.381 | (omitted) |  | (omitted) |  | |
|  | Year 42 | -0.123 | 0.226 | -0.320 | 0.171 | -0.113 | 0.403 | |
|  | Year >=43 | -0.174 | 0.215 | 0.034 | 0.839 | 0.152 | 0.366 | |
| Nursing aide | Year 1 | 0.012 | 0.826 | -0.056 | 0.491 | 0.114 | 0.109 | |
|  | Year 2 | 0.002 | 0.966 | -0.012 | 0.890 | 0.045 | 0.474 | |
|  | Year 3 | -0.069 | 0.218 | -0.103 | 0.208 | 0.011 | 0.867 | |
|  | Year 4 | -0.076 | 0.177 | -0.118 | 0.119 | 0.022 | 0.744 | |
|  | Year 5 | 0.029 | 0.638 | -0.007 | 0.937 | 0.079 | 0.311 | |
|  | Year 6 | 0.012 | 0.828 | -0.062 | 0.444 | 0.122 | 0.119 | |
|  | Year 7 | -0.085 | 0.136 | -0.074 | 0.342 | -0.111 | 0.147 | |
|  | Year 8 | -0.009 | 0.895 | 0.065 | 0.540 | -0.029 | 0.717 | |
|  | Year 9 | -0.084 | 0.139 | 0.017 | 0.842 | -0.077 | 0.309 | |
|  | Year 10 | -0.030 | 0.659 | -0.013 | 0.897 | -0.021 | 0.799 | |
|  | Year 11 | -0.048 | 0.417 | -0.064 | 0.461 | -0.001 | 0.990 | |
|  | Year 12 | -0.046 | 0.450 | -0.106 | 0.245 | 0.017 | 0.806 | |
|  | Year 13 | -0.012 | 0.834 | -0.044 | 0.578 | 0.027 | 0.721 | |
|  | Year 14 | -0.060 | 0.266 | -0.055 | 0.472 | -0.011 | 0.874 | |
|  | Year 15 | -0.048 | 0.412 | -0.066 | 0.447 | 0.009 | 0.895 | |
|  | Year 16 | -0.064 | 0.271 | -0.134 | 0.165 | 0.049 | 0.514 | |
|  | Year 17 | -0.075 | 0.184 | -0.049 | 0.577 | -0.013 | 0.839 | |
|  | Year 18 | -0.033 | 0.624 | -0.040 | 0.693 | 0.062 | 0.301 | |
|  | Year 19 | -0.132 | 0.042 | -0.137 | 0.084 | -0.051 | 0.608 | |
|  | Year 20 | -0.039 | 0.528 | -0.092 | 0.372 | 0.054 | 0.536 | |
|  | Year 21 | -0.040 | 0.495 | -0.081 | 0.384 | 0.009 | 0.899 | |
|  | Year 22 | -0.043 | 0.404 | -0.123 | 0.131 | 0.076 | 0.189 | |
|  | Year 23 | -0.063 | 0.240 | -0.139 | 0.094 | -0.003 | 0.972 | |
|  | Year 24 | -0.032 | 0.609 | -0.108 | 0.243 | 0.019 | 0.796 | |
|  | Year 25 | -0.069 | 0.247 | -0.148 | 0.093 | 0.020 | 0.812 | |
|  | Year 26 | -0.007 | 0.910 | -0.118 | 0.175 | 0.152 | 0.118 | |
|  | Year 27 | -0.065 | 0.335 | -0.072 | 0.383 | -0.006 | 0.945 | |
|  | Year 28 | -0.039 | 0.472 | -0.117 | 0.143 | 0.139 | 0.121 | |
|  | Year 29 | -0.019 | 0.745 | -0.094 | 0.247 | 0.062 | 0.444 | |
|  | Year 30 | -0.035 | 0.574 | -0.036 | 0.720 | 0.049 | 0.604 | |
|  | Year 31 | -0.087 | 0.111 | -0.062 | 0.422 | -0.024 | 0.770 | |
|  | Year 32 | -0.054 | 0.383 | -0.151 | 0.172 | 0.019 | 0.811 | |
|  | Year 33 | -0.056 | 0.324 | -0.100 | 0.293 | -0.112 | 0.165 | |
|  | Year 34 | 0.013 | 0.836 | -0.123 | 0.214 | -0.012 | 0.895 | |
|  | Year 35 | 0.008 | 0.901 | 0.002 | 0.989 | 0.112 | 0.274 | |
|  | Year 36 | 0.036 | 0.561 | 0.054 | 0.679 | -0.014 | 0.865 | |
|  | Year 37 | -0.013 | 0.839 | -0.040 | 0.733 | 0.198 | 0.092 | |
|  | Year 38 | -0.046 | 0.492 | -0.158 | 0.138 | -0.175 | 0.125 | |
|  | Year 39 | 0.026 | 0.728 | 0.057 | 0.610 | -0.034 | 0.839 | |
|  | Year 40 | 0.001 | 0.991 | 0.156 | 0.173 | 0.084 | 0.562 | |
|  | Year 41 | 0.089 | 0.381 | 0.255 | 0.225 | 0.589 | 0.001 | |
|  | Year 42 | -0.101 | 0.285 | 0.107 | 0.627 | 0.356 | 0.031 | |
|  | Year >=43 | -0.080 | 0.257 | -0.085 | 0.545 | -0.268 | 0.087 | |
| Clerk | Year 1 | 0.001 | 0.992 | 0.022 | 0.815 | 0.053 | 0.739 | |
|  | Year 2 | -0.139 | 0.242 | 0.066 | 0.577 | -0.208 | 0.160 | |
|  | Year 3 | -0.168 | 0.160 | -0.070 | 0.467 | -0.202 | 0.168 | |
|  | Year 4 | 0.030 | 0.831 | 0.191 | 0.211 | -0.004 | 0.980 | |
|  | Year 5 | -0.165 | 0.225 | -0.011 | 0.938 | -0.186 | 0.218 | |
|  | Year 6 | 0.040 | 0.727 | 0.235 | 0.012 | -0.038 | 0.779 | |
|  | Year 7 | -0.117 | 0.369 | -0.026 | 0.765 | -0.143 | 0.389 | |
|  | Year 8 | -0.185 | 0.152 | 0.018 | 0.923 | -0.277 | 0.122 | |
|  | Year 9 | -0.038 | 0.791 | 0.089 | 0.436 | -0.018 | 0.918 | |
|  | Year 10 | -0.109 | 0.430 | -0.097 | 0.570 | -0.068 | 0.650 | |
|  | Year 11 | -0.116 | 0.371 | 0.118 | 0.306 | -0.166 | 0.257 | |
|  | Year 12 | -0.111 | 0.424 | -0.083 | 0.535 | -0.062 | 0.689 | |
|  | Year 13 | -0.021 | 0.871 | 0.217 | 0.042 | -0.090 | 0.546 | |
|  | Year 14 | -0.027 | 0.844 | 0.157 | 0.310 | -0.042 | 0.806 | |
|  | Year 15 | -0.145 | 0.343 | 0.153 | 0.259 | -0.293 | 0.099 | |
|  | Year 16 | -0.040 | 0.774 | -0.074 | 0.531 | 0.010 | 0.948 | |
|  | Year 17 | -0.097 | 0.554 | 0.361 | 0.008 | -0.213 | 0.271 | |
|  | Year 18 | -0.024 | 0.892 | -0.078 | 0.659 | 0.083 | 0.623 | |
|  | Year 19 | -0.256 | 0.091 | -0.138 | 0.645 | -0.213 | 0.071 | |
|  | Year 20 | -0.171 | 0.249 | 0.008 | 0.950 | -0.184 | 0.573 | |
|  | Year 21 | -0.125 | 0.330 | 0.171 | 0.246 | -0.264 | 0.057 | |
|  | Year 22 | -0.057 | 0.692 | -0.128 | 0.652 | 0.044 | 0.769 | |
|  | Year 23 | -0.037 | 0.786 | -0.082 | 0.445 | -0.046 | 0.771 | |
|  | Year 24 | -0.216 | 0.126 | 0.043 | 0.699 | -0.402 | 0.013 | |
|  | Year 25 | -0.071 | 0.599 | 0.052 | 0.857 | -0.059 | 0.688 | |
|  | Year 26 | -0.056 | 0.667 | 0.045 | 0.753 | -0.016 | 0.921 | |
|  | Year 27 | -0.051 | 0.730 | -0.012 | 0.898 | -0.001 | 0.996 | |
|  | Year 28 | -0.020 | 0.889 | -0.065 | 0.675 | 0.048 | 0.764 | |
|  | Year 29 | -0.167 | 0.238 | -0.069 | 0.642 | -0.200 | 0.269 | |
|  | Year 30 | 0.026 | 0.855 | 0.120 | 0.398 | -0.034 | 0.873 | |
|  | Year 31 | -0.136 | 0.279 | 0.062 | 0.484 | -0.170 | 0.300 | |
|  | Year 32 | -0.178 | 0.151 | -0.088 | 0.452 | -0.318 | 0.231 | |
|  | Year 33 | -0.212 | 0.126 | -0.197 | 0.645 | -0.352 | 0.032 | |
|  | Year 34 | -0.237 | 0.324 | 0.079 | 0.474 | -0.287 | 0.455 | |
|  | Year 35 | -0.166 | 0.188 | -0.025 | 0.986 | -0.088 | 0.566 | |
|  | Year 36 | -0.357 | 0.196 | (omitted) |  | (omitted) |  | |
|  | Year 37 | -0.035 | 0.818 | -0.364 | 0.001 | -0.028 | 0.848 | |
|  | Year 38 | 0.007 | 0.960 | -0.249 | 0.017 | -0.254 | 0.087 | |
|  | Year 39 | -0.167 | 0.343 | (omitted) |  | -0.352 | 0.015 | |
|  | Year 40 | -0.188 | 0.258 | -0.045 | 0.778 | (omitted) |  | |
|  | Year 41 | 0.002 | 0.994 | (omitted) |  | -0.031 | 0.829 | |
|  | Year 42 | -0.220 | 0.188 | 0.111 | 0.625 | (omitted) |  | |
|  | Year >=43 | 0.069 | 0.737 | 0.187 | 0.100 | 0.341 | 0.043 | |
| Other | Year 1 | -0.004 | 0.960 | 0.126 | 0.447 | 0.014 | 0.853 | |
|  | Year 2 | -0.159 | 0.068 | 0.023 | 0.849 | -0.301 | 0.037 | |
|  | Year 3 | -0.210 | 0.013 | -0.034 | 0.790 | -0.230 | 0.026 | |
|  | Year 4 | -0.245 | 0.008 | -0.085 | 0.530 | -0.243 | 0.018 | |
|  | Year 5 | -0.051 | 0.597 | 0.059 | 0.692 | -0.042 | 0.581 | |
|  | Year 6 | -0.021 | 0.773 | 0.086 | 0.489 | 0.018 | 0.834 | |
|  | Year 7 | -0.195 | 0.038 | -0.104 | 0.464 | -0.179 | 0.071 | |
|  | Year 8 | -0.163 | 0.046 | -0.113 | 0.412 | -0.150 | 0.112 | |
|  | Year 9 | -0.074 | 0.388 | -0.034 | 0.848 | -0.015 | 0.866 | |
|  | Year 10 | -0.208 | 0.043 | -0.185 | 0.235 | -0.107 | 0.339 | |
|  | Year 11 | -0.167 | 0.041 | -0.022 | 0.841 | -0.174 | 0.043 | |
|  | Year 12 | -0.179 | 0.021 | -0.128 | 0.314 | -0.168 | 0.027 | |
|  | Year 13 | -0.078 | 0.398 | 0.115 | 0.472 | -0.133 | 0.126 | |
|  | Year 14 | -0.180 | 0.055 | -0.157 | 0.350 | -0.110 | 0.196 | |
|  | Year 15 | -0.217 | 0.006 | -0.124 | 0.354 | -0.165 | 0.024 | |
|  | Year 16 | -0.060 | 0.507 | -0.031 | 0.837 | -0.033 | 0.722 | |
|  | Year 17 | -0.030 | 0.786 | -0.077 | 0.559 | 0.023 | 0.781 | |
|  | Year 18 | -0.116 | 0.319 | 0.000 | 1.000 | -0.026 | 0.838 | |
|  | Year 19 | -0.181 | 0.041 | -0.136 | 0.255 | -0.041 | 0.731 | |
|  | Year 20 | -0.055 | 0.638 | -0.126 | 0.494 | 0.173 | 0.059 | |
|  | Year 21 | -0.110 | 0.210 | 0.054 | 0.751 | -0.113 | 0.211 | |
|  | Year 22 | -0.223 | 0.014 | -0.023 | 0.862 | -0.274 | 0.033 | |
|  | Year 23 | -0.119 | 0.206 | -0.043 | 0.796 | -0.002 | 0.983 | |
|  | Year 24 | -0.178 | 0.054 | -0.133 | 0.400 | -0.192 | 0.112 | |
|  | Year 25 | -0.174 | 0.121 | -0.089 | 0.628 | -0.176 | 0.235 | |
|  | Year 26 | 0.001 | 0.994 | 0.127 | 0.493 | 0.006 | 0.956 | |
|  | Year 27 | -0.028 | 0.753 | -0.042 | 0.764 | 0.029 | 0.748 | |
|  | Year 28 | 0.275 | 0.078 | 0.372 | 0.035 | 0.349 | 0.216 | |
|  | Year 29 | -0.068 | 0.468 | 0.001 | 0.995 | -0.047 | 0.563 | |
|  | Year 30 | -0.117 | 0.293 | 0.109 | 0.409 | -0.341 | 0.000 | |
|  | Year 31 | 0.002 | 0.989 | 0.277 | 0.478 | -0.141 | 0.124 | |
|  | Year 32 | -0.084 | 0.527 | -0.061 | 0.698 | 0.049 | 0.861 | |
|  | Year 33 | -0.106 | 0.282 | -0.025 | 0.879 | -0.125 | 0.208 | |
|  | Year 34 | 0.088 | 0.584 | 0.122 | 0.449 | 0.348 | 0.633 | |
|  | Year 35 | -0.039 | 0.790 | -0.080 | 0.627 | -0.008 | 0.987 | |
|  | Year 36 | 0.080 | 0.524 | 0.239 | 0.140 | 0.269 | 0.758 | |
|  | Year 37 | -0.093 | 0.501 | 0.279 | 0.043 | -0.115 | 0.354 | |
|  | Year 38 | -0.230 | 0.178 | -0.813 | 0.000 | -0.059 | 0.403 | |
|  | Year 39 | 0.106 | 0.427 | 0.428 | 0.016 | (omitted) |  | |
|  | Year 40 | -0.116 | 0.125 | (omitted) |  | (omitted) |  | |
|  | Year 41 | -0.235 | 0.266 | (omitted) |  | -0.867 | 0.000 | |
|  | Year 42 | -0.082 | 0.397 | 0.302 | 0.202 | -0.114 | 0.067 | |
|  | Year >=43 | -0.221 | 0.372 | -0.469 | 0.402 | (omitted) |  | |
| Gender (Male) |  | -0.107 | 0.000 | -0.131 | 0.000 | -0.087 | 0.000 | |
| Marital Status (Married) |  | -0.014 | 0.008 | -0.009 | 0.336 | -0.011 | 0.159 | |
| Medical, surgical and other specialists per 1,000 population |  | 0.058 | 0.000 | 0.054 | 0.000 | 0.062 | 0.000 | |
| Primary care practitioners per 1,000 population |  | -0.212 | 0.000 | -0.222 | 0.000 | -0.216 | 0.000 | |
| Race - Other (white) |  | 0.030 | 0.000 | 0.048 | 0.000 | 0.024 | 0.037 | |
| Region of employment or residence (New England) |  |  |  |  |  |  |  | |
| Middle Atlantic |  | -0.041 | 0.000 | -0.002 | 0.902 | -0.063 | 0.000 | |
| East North Central |  | -0.107 | 0.000 | -0.097 | 0.000 | -0.110 | 0.000 | |
| West North Central |  | -0.126 | 0.000 | -0.097 | 0.000 | -0.133 | 0.000 | |
| South Atlantic |  | -0.088 | 0.000 | -0.061 | 0.000 | -0.110 | 0.000 | |
| East South Central |  | -0.164 | 0.000 | -0.145 | 0.000 | -0.182 | 0.000 | |
| West South Central |  | -0.075 | 0.000 | -0.063 | 0.003 | -0.091 | 0.000 | |
| Mountain |  | -0.028 | 0.013 | 0.000 | 0.982 | -0.044 | 0.006 | |
| Pacific |  | 0.160 | 0.000 | 0.157 | 0.000 | 0.162 | 0.000 | |
| Work Status (Full-time no-overwork) |  |  |  |  |  |  |  | |
| Part-time |  | -0.079 | 0.000 | -0.083 | 0.000 | -0.067 | 0.000 | |
| Full-time overwork |  | -0.121 | 0.000 | -0.125 | 0.000 | -0.117 | 0.000 | |
| Highest RN/RN-related education^3^ |  |  |  |  |  |  |  | |
| Diploma |  | 0.014 | 0.098 | (omitted) |  | (omitted) |  | |
| Associate |  |  |  | (omitted) |  |  |  | |
| Bachelor |  | 0.048 | 0.000 |  |  | 0.046 | 0.000 | |
| Master’s |  | 0.233 | 0.000 | 0.204 | 0.000 | 0.232 | 0.000 | |
| Work setting (Hospital) |  |  |  |  |  |  |  | |
| Nursing Home |  | -0.152 | 0.000 | -0.146 | 0.000 | -0.140 | 0.000 | |
| Other setting |  | -0.140 | 0.000 | -0.141 | 0.000 | -0.121 | 0.000 | |
| Constant |  | 3.277 | 0.000 | 3.262 | 0.000 | 3.349 | 0.000 | |

Note: * P<0.1, ** P<0.05, *** P<0.01; NA: not applicable; LPN/LVN: Licensed Practical Nurses/Licensed Vocational Nurses

‡ Logarithm form of Registered Nurse (RN) hourly wage; § Heckman’s Sample Selection model: The first-stage equation’s dependent variable was a dichotomous variable indicating working or not. The covariates uniquely included in the first-stage equation (i.e., excluded from the second-stage equation) were other household income, county-level characteristics (uninsurance rate and unemployment rate), age (five categories), student status (full-time, part-time, or no student) and children at home (four categories). Another set of covariates included in both the first-stage equation and the second-stage equation were six categories of prior healthcare employment (manager, LPN/LVN, allied health, nursing aide, clerk, and all other healthcare positions), race, gender, marital status, highest nursing degree, county-level characteristics (primary care practitioners per 1,000 population; and medical, surgical and other specialists per 1,000 population), and indicators for 9 census regions. The second-stage equation’s estimates were presented in this Table S2.; † NSSRN: nationally representative National Sample Survey of Registered Nurses;

^1^ All actively licensed RNs in the NSSRN 2008 public use data file, excluding RNs who resided outside the United States only (i.e., the same as the total population for the first submission). Thus, this “old” total population still included RNs whose initial nursing education was at the diploma or graduate (Master’s or Doctorate) level.

^2^ Experience was calculated by subtracting the year of first RN license from 2008. Additionally, one year was subtracted for RNs who left nursing for one or more years since becoming an RN (unweighted 11.7% of sample), and 0.5 year was subtracted for RNs who are recent graduates and could not have left nursing for one or more years (unweighted 0.9% of sample).

^3^ Bachelor is the reference for the sample of Initial Bachelor degree only.
