# Supplementary material for: The effect of prior healthcare employment on the wages of registered nurses
Source: BMC Health Serv Res. 2016 Aug 19;16:412. doi: 10.1186/s12913-016-1667-0 (PMC4992246; doi:10.1186/s12913-016-1667-0)
Supplement: Additional file 4: Table S4. — Supplemental analyses for Table 1, comparing demographic characteristics between “All sample” and “Working sample,” regarding the number of observations for each covariate. Table S4 consists of six tables below. Table S4-1: Unweighted Total Population (combining Initial Associate and BSN degree). Table S4-2: Unweighted Population (Initial Bachelor degree). Table S4-3: Unweighted Population (Initial Associate degree). Table S4-4: Weighted Total Population (combining Initial Associate and BSN degree). Table S4-5: Weighted Population (Initial Bachelor degree). Table S4-6: Weighted Population (Initial Associate degree). (DOCX 88 kb) [file 12913_2016_1667_MOESM4_ESM.docx]

**Table S4.** Comparison of Demographic Characteristics between “All sample” and “Working sample,” regarding the number of observations for each covariate.

Of note, “All sample” was included in the Heckman model’s first-stage selection equation where the dependent variable was a dichotomous variable indicating whether a subject was working or not as a RN. “Working sample” was included in the Heckman model’s second-stage selection equation where the dependent variable was the logarithm of the RN hourly wage.

Table S4 consists of six tables below.

- Table S4-1: Unweighted Total Population (combining Initial Associate and BSN degree)
- Table S4-2: Unweighted Population (Initial Bachelor degree)
- Table S4-3: Unweighted Population (Initial Associate degree)
- Table S4-4: Weighted Total Population (combining Initial Associate and BSN degree)
- Table S4-5: Weighted Population (Initial Bachelor degree)
- Table S4-6: Weighted Population (Initial Associate degree)

**Table S4-1:** Comparison of Demographic Characteristics between “All sample” and “Working sample” among Unweighted Total Population (combining Initial Associate and BSN degree)

| **Target population** | **Total (Initial Associate and Bachelor degree)** | | | |
| --- | --- | --- | --- | --- |
| **Sample size** | **Un-weighted N=24,136** | | **Un-weighted N=21,225** | |
| **Sample group** | **All sample (selection equation)** | | **Working sample (wage equation)** | |
| **I. Continuous variables** | **Observations** | **Mean (SD)** | **Observations** | **Mean (SD)** |
| **RN hourly wage [$]** |  |  | 21,225 | 30.92 (13.12) |
| **Log of RN hourly wage (Outcome variable)** |  |  | 21,225 | 3.36 (0.36) |
| **RN Experience (in years)** |  |  | 21,225 | 16.79 (10.80) |
| **Medical, surgical and other specialists per 1,000 population** | 24,136 | 1.13 (0.89) | 21,225 | 1.15 (0.90) |
| **Primary care practitioners per 1,000 population** | 24,136 | 0.34 (0.17) | 21,225 | 0.35 (0.17) |
| **Log of other income** | 24,136 | 9.16 (3.54) |  |  |
| **Uninsured rate** | 24,136 | 13.78 (4.14) |  |  |
| **Unemployment rate** | 24,136 | 5.36 (1.62) |  |  |
| **II. Categorical variables** | **Observations** | **Percent (%)** | **Observations** | **Percent (%)** |
| **Prior healthcare position** |  |  |  |  |
| Manager | 461 | (1.9) | 395 | (1.9) |
| LPN/LVN | 4,033 | (16.7) | 3,605 | (17.0) |
| Allied health | 2,849 | (11.8) | 2,548 | (12.0) |
| Nursing aide | 8,470 | (35.1) | 7,506 | (35.4) |
| Clerk | 753 | (3.1) | 661 | (3.1) |
| Other | 1,163 | (4.8) | 1,004 | (4.7) |
| No prior health care job | 6,407 | (26.6) | 5,506 | (25.9) |
| **Gender** |  |  |  |  |
| Male | 1,891 | (7.8) | 1,718 | (8.1) |
| Female | 22,245 | (92.2) | 19,507 | (91.9) |
| **Marital Status** |  |  |  |  |
| Married | 17,900 | (74.2) | 15,684 | (73.9) |
| Single | 6,236 | (25.8) | 5,541 | (26.1) |
| **Race** |  |  |  |  |
| White | 20,426 | (84.6) | 17,868 | (84.2) |
| Other | 3,710 | (15.4) | 3,357 | (15.8) |
| **Work Status** |  |  |  |  |
| Part-time | n/a | n/a | 4,908 | (23.1) |
| Full-time, no overtime | n/a | n/a | 8,756 | (41.3) |
| Full-time, with overtime | n/a | n/a | 7,561 | (35.6) |
| **Work setting** |  |  |  |  |
| Hospital | n/a | n/a | 13,018 | (61.3) |
| Nursing home | n/a | n/a | 1,222 | (5.8) |
| Other setting | n/a | n/a | 6,985 | (32.9) |
| **Highest nursing education** |  |  |  |  |
| Diploma |  |  |  |  |
| Associate degree | 10,747 | (44.5) | 9,553 | (45.0) |
| Bachelor | 10,032 | (41.6) | 8,748 | (41.2) |
| Master’s | 3,357 | (13.9) | 2,924 | (13.8) |
| **Working or not (Selection variable)** |  |  |  |  |
| Working | 21,225 | (87.9) |  |  |
| Not working | 2,911 | (12.1) |  |  |
| **Grouped age** |  |  |  |  |
| <30 | 2,314 | (9.6) | 2,194 | (10.3) |
| 30-39 | 4,964 | (20.6) | 4,532 | (21.4) |
| 40-49 | 6,745 | (28.0) | 6,123 | (28.9) |
| 50-59 | 7,701 | (31.9) | 6,769 | (31.9) |
| >= 60 | 2,412 | (10.0) | 1,607 | (7.6) |
| **Student enrollment** |  |  |  |  |
| Full-time student | 734 | (3.0) | 640 | (3.0) |
| Part-time student | 1,287 | (5.3) | 1,203 | (5.7) |
| Not a student | 22,115 | (91.6) | 19,382 | (91.3) |
| **Children at home** |  |  |  |  |
| Children under 6 yrs | 2,279 | (9.4) | 2,046 | (9.6) |
| All children 6-18 yrs | 6,681 | (27.7) | 6,048 | (28.5) |
| Both under and over 6 yrs | 1,666 | (6.9) | 1,477 | (7.0) |
| No children at home | 13,510 | (56.0) | 11,654 | (54.9) |
| **Urban/Rural** |  |  |  |  |
| Urban | 18,504 | (76.7) | 16,195 | (76.3) |
| Rural | 5,632 | (23.3) | 5,030 | (23.7) |

**Table S4-2:**

Comparison of Demographic Characteristics between “All sample” and “Working sample” among Unweighted Population (Initial Bachelor degree)

| **Target population** | **Initial Bachelor degree** | | | |
| --- | --- | --- | --- | --- |
| **Sample size** | **Un-weighted N=10,345** | | **Un-weighted N=8,992** | |
| **Sample group** | **All sample (selection equation)** | | **Working sample (wage equation)** | |
| **I. Continuous variables** | **Observations** | **Mean (SD)** | **Observations** | **Mean (SD)** |
| **RN hourly wage [$]** |  |  | 8,992 | 32.53 (14.54) |
| **Log of RN hourly wage (Outcome variable)** |  |  | 8,992 | 3.41 (0.37) |
| **RN Experience (in years)** |  |  | 8,992 | 17.52 (11.39) |
| **Medical, surgical and other specialists per 1,000 population** | 10,345 | 1.27 (0.92) | 8,992 | 1.30 (0.93) |
| **Primary care practitioners per 1,000 population** | 10,345 | 0.35 (0.17) | 8,992 | 0.35 (0.17) |
| **Log of other income** | 10,345 | 9.32 (3.53) |  |  |
| **Uninsured rate** | 10,345 | 13.66 (4.22) |  |  |
| **Unemployment rate** | 10,345 | 5.22 (1.56) |  |  |
| **II. Categorical variables** | **Observations** | **Percent (%)** | **Observations** | **Percent (%)** |
| **Prior healthcare position** |  |  |  |  |
| Manager | 136 | (1.3) | 105 | (1.2) |
| LPN/LVN | 564 | (5.5) | 498 | (5.5) |
| Allied health | 1,028 | (9.9) | 906 | (10.1) |
| Nursing aide | 4,563 | (44.1) | 4,010 | (44.6) |
| Clerk | 277 | (2.7) | 247 | (2.8) |
| Other | 539 | (5.2) | 462 | (5.1) |
| No prior health care job | 3,238 | (31.3) | 2,764 | (30.7) |
| **Gender** |  |  |  |  |
| Male | 774 | (7.5) | 691 | (7.7) |
| Female | 9,571 | (92.5) | 8,301 | (92.3) |
| **Marital Status** |  |  |  |  |
| Married | 7,776 | (75.2) | 6,703 | (74.5) |
| Single | 2,569 | (24.8) | 2,289 | (25.5) |
| **Race** |  |  |  |  |
| White | 8,479 | (82.0) | 7,322 | (81.4) |
| Other | 1,866 | (18.0) | 1,670 | (18.6) |
| **Work Status** |  |  |  |  |
| Part-time | n/a | n/a | 2,283 | (25.4) |
| Full-time, no overtime | n/a | n/a | 3,624 | (40.3) |
| Full-time, with overtime | n/a | n/a | 3,085 | (34.3) |
| **Work setting** |  |  |  |  |
| Hospital | n/a | n/a | 5,538 | (61.6) |
| Nursing home | n/a | n/a | 340 | (3.8) |
| Other setting | n/a | n/a | 3,114 | (34.6) |
| **Highest nursing education** |  |  |  |  |
| Diploma |  |  |  |  |
| Associate degree |  |  |  |  |
| Bachelor | 8,023 | (77.6) | 6,990 | (77.7) |
| Master’s | 2,322 | (22.5) | 2,002 | (22.3) |
| **Working or not (Selection variable)** |  |  |  |  |
| Working | 8,992 | (86.9) |  |  |
| Not working | 1,353 | (13.1) |  |  |
| **Grouped age** |  |  |  |  |
| <30 | 1,407 | (13.6) | 1,327 | (14.8) |
| 30-39 | 2,382 | (23.0) | 2,125 | (23.6) |
| 40-49 | 2,770 | (26.8) | 2,473 | (27.5) |
| 50-59 | 2,912 | (28.2) | 2,499 | (27.8) |
| >= 60 | 874 | (8.5) | 568 | (6.3) |
| **Student enrollment** |  |  |  |  |
| Full-time student | 315 | (3.0) | 259 | (2.9) |
| Part-time student | 444 | (4.3) | 415 | (4.6) |
| Not a student | 9,586 | (92.7) | 8,318 | (92.5) |
| **Children at home** |  |  |  |  |
| Children under 6 yrs | 1,265 | (12.2) | 1,114 | (12.4) |
| All children 6-18 yrs | 2,850 | (27.6) | 2,533 | (28.2) |
| Both under and over 6 yrs | 766 | (7.4) | 663 | (7.4) |
| No children at home | 5,464 | (52.8) | 4,682 | (52.1) |
| **Urban/Rural** |  |  |  |  |
| Urban | 8,579 | (82.9) | 7,445 | (82.8) |
| Rural | 1,766 | (17.1) | 1,547 | (17.2) |

**Table S4-3:**

Comparison of Demographic Characteristics between “All sample” and “Working sample” among Unweighted Population (Initial Associate degree)

| **Target population** | **Initial Associate degree** | | | |
| --- | --- | --- | --- | --- |
| **Sample size** | **Un-weighted N=13,791** | | **Un-weighted N=12,223** | |
| **Sample group** | **All sample (selection equation)** | | **Working sample (wage equation)** | |
| **I. Continuous variables** | **Observations** | **Mean (SD)** | **Observations** | **Mean (SD)** |
| **RN hourly wage [$]** |  |  | 12,233 | 29.73 (11.84) |
| **Log of RN hourly wage (Outcome variable)** |  |  | 12,233 | 3.33 (0.35) |
| **RN Experience (in years)** |  |  | 12,233 | 16.25 (10.31) |
| **Medical, surgical and other specialists per 1,000 population** | 13,791 | 1.03 (0.84) | 12,233 | 1.04 (0.85) |
| **Primary care practitioners per 1,000 population** | 13,791 | 0.34 (0.17) | 12,233 | 0.34 (0.17) |
| **Log of other income** | 13,791 | 9.04 (3.55) |  |  |
| **Uninsured rate** | 13,791 | 13.87 (4.07) |  |  |
| **Unemployment rate** | 13,791 | 5.47 (1.65) |  |  |
| **II. Categorical variables** | **Observations** | **Percent (%)** | **Observations** | **Percent (%)** |
| **Prior healthcare position** |  |  |  |  |
| Manager | 325 | (2.4) | 290 | (2.4) |
| LPN/LVN | 3,469 | (25.2) | 3,107 | (25.4) |
| Allied health | 1,821 | (13.2) | 1,642 | (13.4) |
| Nursing aide | 3,907 | (28.3) | 3,496 | (28.6) |
| Clerk | 476 | (3.5) | 414 | (3.4) |
| Other | 624 | (4.5) | 542 | (4.4) |
| No prior health care job | 3,169 | (23.0) | 2,742 | (22.4) |
| **Gender** |  |  |  |  |
| Male | 1,117 | (8.1) | 1,027 | (8.4) |
| Female | 12,674 | (91.9) | 11,206 | (91.6) |
| **Marital Status** |  |  |  |  |
| Married | 10,124 | (73.4) | 8,981 | (73.4) |
| Single | 3,667 | (26.6) | 3,252 | (26.6) |
| **Race** |  |  |  |  |
| White | 11,947 | (86.6) | 10,546 | (86.2) |
| Other | 1,844 | (13.4) | 1,687 | (13.8) |
| **Work Status** |  |  |  |  |
| Part-time | n/a | n/a | 2,625 | (21.5) |
| Full-time, no overtime | n/a | n/a | 5,132 | (42.0) |
| Full-time, with overtime | n/a | n/a | 4,476 | (36.6) |
| **Work setting** |  |  |  |  |
| Hospital | n/a | n/a | 7,480 | (61.2) |
| Nursing home | n/a | n/a | 882 | (7.2) |
| Other setting | n/a | n/a | 3,871 | (31.6) |
| **Highest nursing education** |  |  |  |  |
| Diploma |  |  |  |  |
| Associate degree | 10,747 | (77.9) | 9,553 | (78.1) |
| Bachelor | 2,009 | (14.6) | 1,758 | (14.4) |
| Master’s | 1,035 | (7.5) | 922 | (7.5) |
| **Working or not (Selection variable)** |  |  |  |  |
| Working | 12,233 | (88.7) |  |  |
| Not working | 1,558 | (11.3) |  |  |
| **Grouped age** |  |  |  |  |
| <30 | 907 | (6.6) | 867 | (7.1) |
| 30-39 | 2,582 | (18.7) | 2,407 | (19.7) |
| 40-49 | 3,975 | (28.8) | 3,650 | (29.8) |
| 50-59 | 4,789 | (34.7) | 4,270 | (34.9) |
| >= 60 | 1,538 | (11.2) | 1,039 | (8.5) |
| **Student enrollment** |  |  |  |  |
| Full-time student | 419 | (3.0) | 381 | (3.1) |
| Part-time student | 843 | (6.1) | 788 | (6.4) |
| Not a student | 12,529 | (90.9) | 11,064 | (90.4) |
| **Children at home** |  |  |  |  |
| Children under 6 yrs | 1,014 | (7.4) | 932 | (7.6) |
| All children 6-18 yrs | 3,831 | (27.8) | 3,515 | (28.7) |
| Both under and over 6 yrs | 900 | (6.5) | 814 | (6.7) |
| No children at home | 8,046 | (58.3) | 6,972 | (57.0) |
| **Urban/Rural** |  |  |  |  |
| Urban | 9,925 | (72.0) | 8,750 | (71.5) |
| Rural | 3,866 | (28.0) | 3,483 | (28.5) |

**Table S4-4:**

Comparison of Demographic Characteristics between “All sample” and “Working sample” among Weighted Total Population (combining Initial Associate and BSN degree)

| **Target population** | **Total (Initial Associate and Bachelor degree)** | | | |
| --- | --- | --- | --- | --- |
| **Un-weighted sample size** | **Un-weighted N=24,136** | | **Un-weighted N=21,225** | |
| **Weighted sample size** | **Weighted N=2,242,238** | | **Weighted N=1,949,625** | |
| **Sample group** | **All sample (selection equation)** | | **Working sample (wage equation)** | |
| **I. Continuous variables** | **Weighted sample n** | **Mean (SD)** | **Weighted sample n** | **Mean (SD)** |
| **RN hourly wage [$]** |  |  | 1,949,625 | 31.14 (12.68) |
| **Log of RN hourly wage (Outcome variable)** |  |  | 1,949,625 | 3.37 (0.36) |
| **RN Experience (in years)** |  |  | 1,949,625 | 15.85 (10.68) |
| **Medical, surgical and other specialists per 1,000 population** | 2,242,238 | 1.14 (0.87) | 1,949,625 | 1.16 (0.88) |
| **Primary care practitioners per 1,000 population** | 2,242,238 | 0.32 (0.15) | 1,949,625 | 0.32 (0.15) |
| **Log of other income** | 2,242,238 | 9.18 (3.56) |  |  |
| **Uninsured rate** | 2,242,238 | 14.45 (4.44) |  |  |
| **Unemployment rate** | 2,242,238 | 5.72 (1.53) |  |  |
| **II. Categorical variables** | **Weighted sample n** | **Percent (%)** | **Weighted sample n** | **Percent (%)** |
| **Prior healthcare position** |  |  |  |  |
| Manager | 40,583 | (1.8) | 33,694 | (1.7) |
| LPN/LVN | 353,334 | (15.8) | 311,253 | (16.0) |
| Allied health | 267,431 | (11.9) | 237,609 | (12.2) |
| Nursing aide | 780,764 | (34.8) | 682,037 | (35.0) |
| Clerk | 72,290 | (3.2) | 64,315 | (3.3) |
| Other | 105,368 | (4.7) | 90,257 | (4.6) |
| No prior health care job | 622,469 | (27.8) | 530,461 | (27.2) |
| **Gender** |  |  |  |  |
| Male | 163,941 | (7.3) | 148,476 | (7.6) |
| Female | 2,078,297 | (92.7) | 1,801,149 | (92.4) |
| **Marital Status** |  |  |  |  |
| Married | 1,666,373 | (74.3) | 1,441,195 | (73.9) |
| Single | 575,865 | (25.7) | 508,430 | (26.1) |
| **Race** |  |  |  |  |
| White | 1,844,263 | (82.3) | 1,588,066 | (81.5) |
| Other | 397,975 | (17.8) | 361,559 | (18.6) |
| **Work Status** |  |  |  |  |
| Part-time | n/a | n/a | 456,736 | (23.4) |
| Full-time, no overtime | n/a | n/a | 802,250 | (41.2) |
| Full-time, with overtime | n/a | n/a | 690,639 | (35.4) |
| **Work setting** |  |  |  |  |
| Hospital | n/a | n/a | 1,241,302 | (63.7) |
| Nursing home | n/a | n/a | 102,345 | (5.3) |
| Other setting | n/a | n/a | 605,978 | (31.1) |
| **Highest nursing education** |  |  |  |  |
| Diploma |  |  |  |  |
| Associate degree | 1,022,483 | (45.6) | 899,364 | (46.1) |
| Bachelor | 934,706 | (41.7) | 807,136 | (41.4) |
| Master’s | 285,049 | (12.7) | 243,124 | (12.5) |
| **Working or not (Selection variable)** |  |  |  |  |
| Working | 1,949,625 | (87.0) |  |  |
| Not working | 292,613 | (13.1) |  |  |
| **Grouped age** |  |  |  |  |
| <30 | 245,879 | (11.0) | 234,020 | (12.0) |
| 30-39 | 521,901 | (23.3) | 474,743 | (24.4) |
| 40-49 | 637,952 | (28.5) | 572,198 | (29.4) |
| 50-59 | 625,271 | (27.9) | 538,712 | (27.6) |
| >= 60 | 211,236 | (9.4) | 129,952 | (6.7) |
| **Student enrollment** |  |  |  |  |
| Full-time student | 73,393 | (3.3) | 64,701 | (3.3) |
| Part-time student | 122,519 | (5.5) | 114,400 | (5.9) |
| Not a student | 2,046,325 | (91.3) | 1,770,523 | (90.8) |
| **Children at home** |  |  |  |  |
| Children under 6 yrs | 239,722 | (10.7) | 214,155 | (11.0) |
| All children 6-18 yrs | 648,426 | (28.9) | 579,336 | (29.7) |
| Both under and over 6 yrs | 170,475 | (7.6) | 150,352 | (7.7) |
| No children at home | 1,183,615 | (52.8) | 1,005,781 | (51.6) |
| **Urban/Rural** |  |  |  |  |
| Urban | 1,819,852 | (81.2) | 1,574,776 | (80.8) |
| Rural | 422,386 | (18.8) | 374,848 | (19.2) |

**Table S4-5:**

Comparison of Demographic Characteristics between “All sample” and “Working sample” among Weighted Population (Initial Bachelor degree)

| **Target population** | **Initial Bachelor degree** | | | |
| --- | --- | --- | --- | --- |
| **Un-weighted sample size** | **Un-weighted N=10,345** | | **Un-weighted N=8,992** | |
| **Weighted sample size** | **Weighted N=945,429** | | **Weighted N=812,458** | |
| **Sample group** | **All sample (selection equation)** | | **Working sample (wage equation)** | |
| **I. Continuous variables** | **Weighted sample n** | **Mean (SD)** | **Weighted sample n** | **Mean (SD)** |
| **RN hourly wage [$]** |  |  | 812,458 | 32.90 (13.65) |
| **Log of RN hourly wage (Outcome variable)** |  |  | 812,458 | 3.43 (0.36) |
| **RN Experience (in years)** |  |  | 812,458 | 16.44 (11.19) |
| **Medical, surgical and other specialists per 1,000 population** | 945,429 | 1.28 (0.91) | 812,458 | 1.30 (0.92) |
| **Primary care practitioners per 1,000 population** | 945,429 | 0.32 (0.15) | 812,458 | 0.32 (0.15) |
| **Log of other income** | 945,429 | 9.34 (3.53) |  |  |
| **Uninsured rate** | 945,429 | 14.41 (4.57) |  |  |
| **Unemployment rate** | 945,429 | 5.58 (1.42) |  |  |
| **II. Categorical variables** | **Weighted sample n** | **Percent (%)** | **Weighted sample n** | **Percent (%)** |
| **Prior healthcare position** |  |  |  |  |
| Manager | 12,056 | (1.3) | 8,809 | (1.1) |
| LPN/LVN | 46,782 | (5.0) | 40,783 | (5.0) |
| Allied health | 94,316 | (10.0) | 81,849 | (10.1) |
| Nursing aide | 406,120 | (43.0) | 351,355 | (43.3) |
| Clerk | 25,453 | (2.7) | 22,881 | (2.8) |
| Other | 46,869 | (5.0) | 39,890 | (4.9) |
| No prior health care job | 313,832 | (33.2) | 266,892 | (32.9) |
| **Gender** |  |  |  |  |
| Male | 64,904 | (6.9) | 57,484 | (7.1) |
| Female | 880,525 | (93.1) | 754,974 | (92.9) |
| **Marital Status** |  |  |  |  |
| Married | 711,997 | (75.3) | 605,404 | (74.5) |
| Single | 233,432 | (24.7) | 207,054 | (25.5) |
| **Race** |  |  |  |  |
| White | 741,759 | (78.5) | 629,043 | (77.4) |
| Other | 203,670 | (21.5) | 183,416 | (22.6) |
| **Work Status** |  |  |  |  |
| Part-time | n/a | n/a | 208,498 | (25.7) |
| Full-time, no overtime | n/a | n/a | 327,744 | (40.3) |
| Full-time, with overtime | n/a | n/a | 276,217 | (34.0) |
| **Work setting** |  |  |  |  |
| Hospital | n/a | n/a | 528,502 | (65.1) |
| Nursing home | n/a | n/a | 27,304 | (3.4) |
| Other setting | n/a | n/a | 256,653 | (31.6) |
| **Highest nursing education** |  |  |  |  |
| Diploma |  |  |  |  |
| Associate degree |  |  |  |  |
| Bachelor | 749,974 | (79.3) | 647,623 | (79.7) |
| Master’s | 195,455 | (20.7) | 164,836 | (20.3) |
| **Working or not (Selection variable)** |  |  |  |  |
| Working | 812,458 | (85.9) |  |  |
| Not working | 132,970 | (14.1) |  |  |
| **Grouped age** |  |  |  |  |
| <30 | 142,898 | (15.1) | 135,019 | (16.6) |
| 30-39 | 244,912 | (25.9) | 216,300 | (26.6) |
| 40-49 | 256,511 | (27.1) | 225,284 | (27.7) |
| 50-59 | 229,908 | (24.3) | 192,340 | (23.7) |
| >= 60 | 71,200 | (7.5) | 43,516 | (5.4) |
| **Student enrollment** |  |  |  |  |
| Full-time student | 31,557 | (3.3) | 26,180 | (3.2) |
| Part-time student | 41,278 | (4.4) | 38,671 | (4.8) |
| Not a student | 872,593 | (92.3) | 747,608 | (92.0) |
| **Children at home** |  |  |  |  |
| Children under 6 yrs | 127,984 | (13.5) | 111,953 | (13.8) |
| All children 6-18 yrs | 271,476 | (28.7) | 237,119 | (29.2) |
| Both under and over 6 yrs | 79,535 | (8.4) | 67,807 | (8.4) |
| No children at home | 466,433 | (49.3) | 395,580 | (48.7) |
| **Urban/Rural** |  |  |  |  |
| Urban | 827,460 | (87.5) | 709,021 | (87.3) |
| Rural | 117,969 | (12.5) | 103,437 | (12.7) |

**Table S4-6:**

Comparison of Demographic Characteristics between “All sample” and “Working sample” among Weighted Population (Initial Associate degree)

| **Target population** | **Initial Associate degree** | | | |
| --- | --- | --- | --- | --- |
| **Un-weighted sample size** | **Un-weighted N=13,791** | | **Un-weighted N=12,223** | |
| **Weighted sample size** | **Weighted N=1,296,809** | | **Weighted N=1,137,166** | |
| **Sample group** | **All sample (selection equation)** | | **Working sample (wage equation)** | |
| **I. Continuous variables** | **Weighted sample n** | **Mean (SD)** | **Weighted sample n** | **Mean (SD)** |
| **RN hourly wage [$]** |  |  | 1,137,166 | 29.88 (11.78) |
| **Log of RN hourly wage (Outcome variable)** |  |  | 1,137,166 | 3.33 (0.35) |
| **RN Experience (in years)** |  |  | 1,137,166 | 15.43 (10.29) |
| **Medical, surgical and other specialists per 1,000 population** | 1,296,809 | 1.03 (0.83) | 1,137,166 | 1.05 (0.84) |
| **Primary care practitioners per 1,000 population** | 1,296,809 | 0.32 (0.15) | 1,137,166 | 0.32 (0.15) |
| **Log of other income** | 1,296,809 | 9.06 (3.57) |  |  |
| **Uninsured rate** | 1,296,809 | 14.49 (4.35) |  |  |
| **Unemployment rate** | 1,296,809 | 5.83 (1.59) |  |  |
| **II. Categorical variables** | **Weighted sample n** | **Percent (%)** | **Weighted sample n** | **Percent (%)** |
| **Prior healthcare position** |  |  |  |  |
| Manager | 28,527 | (2.2) | 24,885 | (2.2) |
| LPN/LVN | 306,553 | (23.6) | 270,470 | (23.8) |
| Allied health | 173,114 | (13.4) | 155,760 | (13.7) |
| Nursing aide | 374,643 | (28.9) | 330,682 | (29.1) |
| Clerk | 46,836 | (3.6) | 41,434 | (3.6) |
| Other | 58,499 | (4.5) | 50,367 | (4.4) |
| No prior health care job | 308,637 | (23.8) | 263,568 | (23.2) |
| **Gender** |  |  |  |  |
| Male | 99,037 | (7.6) | 90,992 | (8.0) |
| Female | 1,197,772 | (92.4) | 1,046,175 | (92.0) |
| **Marital Status** |  |  |  |  |
| Married | 954,376 | (73.6) | 835,791 | (73.5) |
| Single | 342,433 | (26.4) | 301,376 | (26.5) |
| **Race** |  |  |  |  |
| White | 1,102,504 | (85.0) | 959,023 | (84.3) |
| Other | 194,305 | (15.0) | 178,143 | (15.7) |
| **Work Status** |  |  |  |  |
| Part-time | n/a | n/a | 248,238 | (21.8) |
| Full-time, no overtime | n/a | n/a | 474,507 | (41.7) |
| Full-time, with overtime | n/a | n/a | 414,422 | (36.4) |
| **Work setting** |  |  |  |  |
| Hospital | n/a | n/a | 712,800 | (62.7) |
| Nursing home | n/a | n/a | 75,041 | (6.6) |
| Other setting | n/a | n/a | 349,325 | (30.7) |
| **Highest nursing education** |  |  |  |  |
| Diploma |  |  |  |  |
| Associate degree | 1,022,483 | (78.9) | 899,364 | (79.1) |
| Bachelor | 184,732 | (14.3) | 159,513 | (14.0) |
| Master’s | 89,594 | (6.9) | 78,288 | (6.9) |
| **Working or not (Selection variable)** |  |  |  |  |
| Working | 1,137,166 | (87.7) |  |  |
| Not working | 159,643 | (12.3) |  |  |
| **Grouped age** |  |  |  |  |
| <30 | 102,981 | (7.9) | 99,001 | (8.7) |
| 30-39 | 276,989 | (21.4) | 258,443 | (22.7) |
| 40-49 | 381,441 | (29.4) | 346,914 | (30.5) |
| 50-59 | 395,364 | (30.5) | 346,372 | (30.5) |
| >= 60 | 140,036 | (10.8) | 86,436 | (7.6) |
| **Student enrollment** |  |  |  |  |
| Full-time student | 41,836 | (3.2) | 38,522 | (3.4) |
| Part-time student | 81,241 | (6.3) | 75,729 | (6.7) |
| Not a student | 1,173,732 | (90.5) | 1,022,915 | (90.0) |
| **Children at home** |  |  |  |  |
| Children under 6 yrs | 111,737 | (8.6) | 102,202 | (9.0) |
| All children 6-18 yrs | 376,950 | (29.1) | 342,217 | (30.1) |
| Both under and over 6 yrs | 90,940 | (7.0) | 82,546 | (7.3) |
| No children at home | 717,182 | (55.3) | 610,201 | (53.7) |
| **Urban/Rural** |  |  |  |  |
| Urban | 992,392 | (76.5) | 865,755 | (76.1) |
| Rural | 304,417 | (23.5) | 271,411 | (23.9) |
